# A multi-commodity network model for optimal quantum reversible circuit synthesis

**DOI:** 10.1371/journal.pone.0253140

**Published:** 2021-06-22

**Authors:** Jihye Jung, In-Chan Choi

**Affiliations:** Quantum Machine Learning Laboratory, School of Industrial Management Engineering, Korea University, Seoul, Republic of Korea; Vellore Institute of Technology: VIT University, INDIA

## Abstract

Quantum computing is a newly emerging computing environment that has recently attracted intense research interest in improving the output fidelity, fully utilizing its high computing power from both hardware and software perspectives. In particular, several attempts have been made to reduce the errors in quantum computing algorithms through the efficient synthesis of quantum circuits. In this study, we present an application of an optimization model for synthesizing quantum circuits with minimum implementation costs to lower the error rates by forming a simpler circuit. Our model has a unique structure that combines the arc-subset selection problem with a conventional multi-commodity network flow model. The model targets the circuit synthesis with multiple control Toffoli gates to implement Boolean reversible functions that are often used as a key component in many quantum algorithms. Compared to previous studies, the proposed model has a unifying yet straightforward structure for exploiting the operational characteristics of quantum gates. Our computational experiment shows the potential of the proposed model, obtaining quantum circuits with significantly lower quantum costs compared to prior studies. The proposed model is also applicable to various other fields where reversible logic is utilized, such as low-power computing, fault-tolerant designs, and DNA computing. In addition, our model can be applied to network-based problems, such as logistics distribution and time-stage network problems.

## Introduction

Quantum computing is a next-generation computing paradigm based on the uncertainty 2 principle underlying quantum mechanics. This new technology has recently attracted attention owing to its high computing power compared to classic computing environments. Quantum computing enables efficient calculation for certain difficult nondeterministic polynomial time (NP) problems such as prime factorization and discrete logarithms [1].

The most significant feature of a quantum computer, differing from a traditional computer, is an information unit called a *quantum bit* or *qubit*. Qubits are based on quantum superposition and quantum entanglement, which are fundamental properties in quantum mechanics. Compared to *bits* in a traditional computing environment, qubits can contain an exponentially large amount of information even with the same number of information units.

Considerable research has been conducted to enhance the practicality of quantum computing in terms of hardware [2, 3] and software. In particular, a wide range of research has been proposed regarding software, from basic to applicational quantum algorithms. For example, basic quantum algorithms include Grover’s algorithm [4] for an unstructured search, Shor’s algorithm [5] for integer factorization, and the Harrow–Hassidim–Lloyd algorithm [6] for simultaneous linear systems. Some applicational algorithms include a quantum genetic algorithm [7], quantum support vector machine [8], quantum principal component analysis [9], and quantum reinforcement learning [10]. Research has also been conducted on applying quantum computing environments to real-world problems such as chemistry [11] and data science [12].

Despite the advantages of quantum computing, computational accuracy is still insufficient for practical use. The major factors that cause computational errors are the qubit and quantum gates. A qubit is implemented using elementary particles such as electrons and photons that maintain stability for only a short period of time [13]. A quantum gate applies a specific operation on the qubit, and this process is implemented as physical stimuli such as a pulse wave. The more often these stimuli are applied to the qubit, the less stable the qubit becomes, increasing the computational error. To overcome the intrinsic hardware issues, research attempts have been made in terms of software development. As a part of these attempts, many studies have been conducted on the design of efficient circuits for quantum computing.

Various metrics for evaluating the circuit efficiency for quantum computing have been proposed. These metrics include the number of gates, computational speed, quantum cost, qubit interaction cost, number of auxiliary qubits, and circuit depth [14]. The quantum cost refers to the total number of basic quantum gates required to implement a logical gate in the quantum computing environment. Thus, it indicates the actual implementation cost of a logic gate or a circuit. Several studies have also been conducted on cost systems for various logic gates and quantum circuits [15, 16].

Based on the presented metrics, various studies have developed methodologies for an efficient circuit synthesis of reversible functions in the quantum computing environment. Most early studies present methodologies based on observations and preconfigured circuit libraries. Methodologies for large-sized reversible functions have been proposed using a template matching approach that exploits a library of small-scale circuits [17–19]. Research has also been conducted to perform post-optimization on the synthesized circuit through a relocation algorithm [20, 21]. However, these methodologies do not guarantee the optimality of the synthesized circuit.

A circuit synthesis methodology based on the systematic searching method has also been proposed. An algorithm to minimize the gate count through a decomposition of a Boolean reversible logic is proposed based on the cycle representation of a given logic [22]. A heuristic algorithm to improve the quantum cost has been considered, which uses the Reed–Muller decomposition to express a binary reversible function as the sum of the products of binary variables [23, 24]. A neighbor searching scheme on exclusive-sum-of-products based reversible logic has also been proposed [25]. A methodology based on binary decision diagrams was developed to handle relatively sizeable reversible circuits [26, 27, 28]. A synthesis algorithm based on the functional decision diagrams and dependency diagrams is also presented [29]. A circuit synthesis using the A* algorithm, a searching scheme based on the shortest path in the search graph, has also been proposed [30]. Most of these heuristic-based approaches can be applied to relatively large-scale problems. However, such algorithms are not guaranteed to obtain optimal results and present challenges when additional constraints are considered. Some post-optimization algorithms have been proposed [31–34] to overcome the shortcomings of the heuristic-based approach.

A methodology was also developed to guarantee the optimality in the number of gates through the satisfiability problem [35]; however, the problem size that the proposed methodology can solve is limited. Furthermore, the number of gates is insufficient to represent a practical cost system in implementing reversible circuits in a quantum computing environment. Several approaches have been presented to search for near-optimal circuits based on evolutionary algorithms such as adaptive genetic algorithms [36] and genetic programming [37]. However, owing to the inherent characteristics of metaheuristics, they cannot guarantee the optimality of the solution. In addition, consideration of additional constraints and objective functions is difficult. In our previous study, we proposed an optimization model to obtain a reversible circuit of the minimal quantum cost [38]. The model uses a complicated external function based on the gate pattern, and hence, the size of the problem that the model can handle is limited to a small scale.

In this study, by accommodating the complex external functions into the model, the proposed model is far more structured compared to our previous approach [38]. For this improvement, we adopt a well-known multi-commodity network flow problem to provide the basic framework for the improved model. Since the 1960s, various studies have applied their designed models to this problem [39]. For famous large-scale optimization techniques such as column generation [40], Benders decomposition [41], and Dantzig–Wolfe decomposition [42], the multi-commodity network flow problem is considered a best-practice example because of its well-defined constraint structure. Several solution methodologies have also been proposed for multi-commodity network flow models such as the parallelized cost decomposition algorithm [43] and the cost reoptimization algorithm [44]. Various studies have been conducted to apply multi-commodity network flow models to practical problems. For example, the model has been applied to fields such as transportation [45], scheduling [46], and production planning [47].

This study is aimed at the development of a novel optimization-based approach for a circuit synthesis of reversible functions with an optimal quantum cost as a methodology in a more robust fashion. Our study is different from previous studies in that the proposed model can evaluate the optimality of a solution and is free to consider additional constraints and objective functions. These differences arise because the proposed methodology is based on an optimization model. Specifically, the proposed model is based on an extended version of the multi-commodity network flow model, which is combined with an arc selection problem of the underlying network. Because the proposed model has a straightforward structure based on the existing well-known problems, it shows better computational results and has the advantage of being a versatile model that can consider additional constraints or be applied to other fields. Quantum algorithms generally adopt a Boolean reversible logic as a key element that often represents the problem to solve [48]. Accordingly, various types of reversible logic need to be circuited. Motivated by this need, our research presents an efficient synthesis methodology of circuits for Boolean reversible logic. A library of logic gates, referred to as a multiple control Toffoli gate, is used for the circuit representation.

### Contributions

This research has two major contributions. First, our optimization-based approach on quantum reversible circuit synthesis is a novel attempt with high robustness from the perspective of reversible circuit synthesis and quantum computing.

Compared to previous model-based studies that minimize the number of gates, our model handles the objective that minimizes the quantum cost of reversible circuits, which implies an actual implementation cost required in a quantum computing environment. Additional constraints can also be freely added to the model according to the user’s purposes or technical issues. In addition, our optimization-based approach guarantees the optimality of the solution and can thus be utilized as the baseline methodology for evaluating circuit synthesis heuristics. Second, we propose an optimization model based on an extension of the multi-commodity network flow model that uniquely appears in the target problem. Compared to a conventional multi-commodity network flow model, where the network cost is defined for each unit flow, the network cost of our model is defined according to the subset of selected arcs. As mentioned earlier, the multi-commodity network flow model is a well-known model with rich theoretical and applicational studies, which allows us to continue further research on the proposed model. Furthermore, the proposed model yields significantly better computational results compared to previous studies. The optimal circuits presented in this study can also be exploited as building blocks to synthesize large-scale circuits.

Furthermore, the proposed model has high applicability to other areas. The reversible circuit synthesis methodology presented in this study can be applied not only to quantum computing but also to other fields that exploit reversible logic, such as low-power computing, fault-tolerant design, nanotechnology, DNA computing, and optical computing [49]. The proposed model, which is an extended form of a multi-commodity network flow model, can also be applied to conventional network problems such as a distribution and time-stage network.

The rest of this paper is organized as follows. In the *Materials and methods* section, we introduce the background concepts and terminologies used in this paper. A detailed description of the target problem, referred to as the quantum reversible circuit synthesis (QRCS) problem, is presented in the following section. The mathematical model for the target problem is described in Section 4. Section 5 shows the computational results of the proposed optimization model on the benchmark datasets. Section 6 presents some concluding remarks regarding this study and a direction for future research.

The rest of this paper is organized as follows. In Section 2, we introduce the background concepts and terminologies used in this paper. A detailed description of the target problem, referred to as the quantum reversible circuit synthesis (QRCS) problem, is presented in the following section. The mathematical model for the target problem is described in Section 4. Section 5 shows the computational results of the proposed optimization model on the benchmark datasets. Section 6 presents some concluding remarks regarding this study and a direction for future research.

## Materials and methods

### Background concepts and terminologies

This subsection is largely divided into four parts: quantum computing, Boolean reversible functions, reversible logic gates, and the multi-commodity network flow model. Here, we describe the basic concepts and terminologies used in each topic, as well as the mathematical notation.

#### Quantum computing

A *quantum bit* or *qubit* is an elementary unit used in the quantum computing environment. It is often physically implemented with a quantum system such as an electron, ion, or photon. When multiple qubits are considered a single entity, it is called a *qubit register*. A single qubit saves the probabilistic information of one of the two deterministic states measured when one observes the qubit. These two pure states are called *computational basis states*.

**Definition 1**. A single qubit retains *two computational basis states*(*CBSs*), a 0 state, and a 1 state, which are respectively represented as |0〉 and |1〉, following a bra-ket notation. Both CBSs can also be denoted by two-dimensional unit vectors, as shown in Eq (1).

$$|0\rangle ={\left[1\;0\right]}^{T},\;|1\rangle ={\left[0\;1\right]}^{T}$$ (1a)

$$|00\rangle ={\left[1\;0\;0\;0\right]}^{T},\;|01\rangle ={\left[0\;1\;0\;0\right]}^{T},\;|10\rangle ={\left[0\;0\;1\;0\right]}^{T},\;|11\rangle ={\left[0\;0\;0\;1\right]}^{T}$$ (1b)

$$|0\cdots 00\rangle ={\left[1\;0\;0\cdots 0\right]}^{T},\;|0\cdots 01\rangle ={\left[0\;1\;0\cdots 0\right]}^{T},\;\cdots ,\;|1\cdots 11\rangle ={\left[0\;0\cdots 0\;1\right]}^{T}$$ (1c)

The notation in Definition 1 can be extended to a qubit register. In a 2-qubit system, the total number of combinations of measurable states is 2^2^ = 4 because both qubits have two CBSs each. In this case, the qubit register has a total of four CBSs: |00〉, |01〉, |10〉, and |11〉. Each binary digit corresponds to the states of the qubit. These CBSs can also be represented as a set of four-dimensional vectors as shown in Eq (1b). For the generalized case of a qubit register with *N* qubits, a total of 2^*N*^ CBSs are formed: |0⋯00〉, |0⋯01〉, ⋯, |1⋯10〉, |1⋯11〉. The binary sequence in the bra-ket notations is composed of *N* digits, which correspond to each of *N* qubits. Eq (1c) shows that each CBS can also be represented as a 2^*N*^ dimensional unit vector.

As mentioned earlier, the qubit saves the probabilistic information of multiple CBSs simultaneously. All of these possible states that a qubit system can represent are called *quantum states*.

**Definition 2**. A *quantum state* |*ψ*〉 of a qubit or a qubit register is the linear combination of all CBSs with complex scalars $\alpha_{i}\in C$, ∀*i* = 1, ⋯, 2^*N*^. Eq 2a shows the general form of a quantum state of *N*-qubit registers.

$$|\psi \rangle =\alpha_{1}|0\cdots 00\rangle +\cdots +\alpha_{2^{N}}|1\cdots 11\rangle$$ (2a)

$$\sum_{i=1}^{2^{N}}|\alpha_{i}{|}^{2}=1$$ (2b)

The squared Euclidean norm of each coefficient implies the measurement probability of the corresponding CBS when one observes a qubit or a qubit register. Therefore, Eq (2b) implies that the sum of all measurement probabilities equals 1.

To change the given quantum state to a desired state, a sequence of *basic quantum gates* is adopted and a quantum algorithm is composed.

**Definition 3**. A *basic quantum gate* is a unit device that physically realizes a unitary operation on a target qubit. The unitary operation is represented as a unitary matrix *U* of size 2^*N*^ × 2^*N*^ in an *N*-qubit system, where *UU*^†^ = *I* when *U*^†^ is the conjugate transpose of *U*.

*Example 1*. Eqs (3a) and (3b) show an algebraic operation of a Pauli *X* gate, a well-known basic quantum gate. As presented in Eq (3a), a Pauli *X* gate is represented as a 2 × 2 unitary matrix *U*_*X*_. It should be noted that *U*_*X*_ is a permutation matrix. In Eq (3b), a Pauli *X* gate conducts a unitary transformation *U*_*X*_ on a single qubit quantum state |*ψ*〉 = *α*_1_|0〉 + *α*_2_|1〉 for $\alpha_{1},\alpha_{2}\in C$. The result of the transformation shows that the probabilities to observe |0〉 and |1〉 are exchanged with each other.

$$U_{X}=[\begin{array}{cc} 0 & 1\\ 1 & 0 \end{array}],\;U_{X}U_{X}^{†}=U_{X}U_{X}=I$$ (3a)

$$U_{X}|\psi \rangle =\alpha_{1}U_{X}|0\rangle +\alpha_{2}U_{X}|1\rangle =\alpha_{1}U_{X}[\begin{array}{c} 1\\ 0 \end{array}]+\alpha_{2}U_{X}[\begin{array}{c} 0\\ 1 \end{array}]=\alpha_{1}[\begin{array}{c} 0\\ 1 \end{array}]+\alpha_{2}[\begin{array}{c} 1\\ 0 \end{array}]=\alpha_{2}|0\rangle +\alpha_{1}|1\rangle$$ (3b)

Various other quantum basic gates, such as the Hadamard gate and phase shift gates, are also frequently used in quantum computing. Some particular basic quantum gates, called *controlled gates*, such as controlled-*X* gates and controlled-*V* gates, operate jointly on multiple qubits.

**Definition 4**. A *controlled gate* is a type of basic quantum gate composed of a control bit and a target bit. In particular, a target bit is assigned with a basic quantum gate for a single qubit. A controlled gate activates the assigned basic quantum gate on a qubit corresponding to a target bit only when the qubit corresponding to the control bit is in state 1.

*Example 2*. A controlled gate with a Pauli *X* gate on its target bit is particularly called a *CNOT gate*(*controlled-NOT gate*). This example presents how a CNOT gate works on a 2-qubit system. Assume that a control bit is located on the first qubit and the target bit is located on the second qubit. In Eq (4a), a CNOT gate is represented as a 4 × 4 unitary matrix *U*_*CNOT*_. It should be noted that *U*_*CNOT*_ is a permutation matrix. Eq (4b) shows an algebraic operation of a CNOT gate on a quantum state |*ψ*〉 = *α*_1_|00〉 + *α*_2_|01〉 + *α*_3_|10〉 + *α*_4_|11〉 for $\alpha_{1},\alpha_{2},\alpha_{3},\alpha_{4}\in C$. The result shows that the probabilities of observing |10〉 and |11〉 are exchanged with each other.

$$U_{CNOT}=[\begin{array}{cccc} 1 & 0 & 0 & 0\\ 0 & 1 & 0 & 0\\ 0 & 0 & 0 & 1\\ 0 & 0 & 1 & 0 \end{array}],\;U_{CNOT}U_{CNOT}^{†}=U_{CNOT}U_{CNOT}=I$$ (4a)

$$\begin{array}{ll} U_{CNOT}|\psi \rangle & =\alpha_{1}U_{CNOT}|00\rangle +\alpha_{2}U_{CNOT}|01\rangle +\alpha_{3}U_{CNOT}|10\rangle +\alpha_{4}U_{CNOT}|11\rangle \\ & =\alpha_{1}U_{CNOT}[\begin{array}{c} 1\\ 0\\ 0\\ 0 \end{array}]+\alpha_{2}U_{CNOT}[\begin{array}{c} 0\\ 1\\ 0\\ 0 \end{array}]+\alpha_{3}U_{CNOT}[\begin{array}{c} 0\\ 0\\ 1\\ 0 \end{array}]+\alpha_{4}U_{CNOT}[\begin{array}{c} 0\\ 0\\ 0\\ 1 \end{array}]\\ & =\alpha_{1}[\begin{array}{c} 1\\ 0\\ 0\\ 0 \end{array}]+\alpha_{2}[\begin{array}{c} 0\\ 1\\ 0\\ 0 \end{array}]+\alpha_{3}[\begin{array}{c} 0\\ 0\\ 0\\ 1 \end{array}]+\alpha_{4}[\begin{array}{c} 0\\ 0\\ 1\\ 0 \end{array}]\\ & =\alpha_{1}|00\rangle +\alpha_{2}|01\rangle +\alpha_{4}|10\rangle +\alpha_{3}|11\rangle \end{array}$$ (4b)

**Fig 1:**
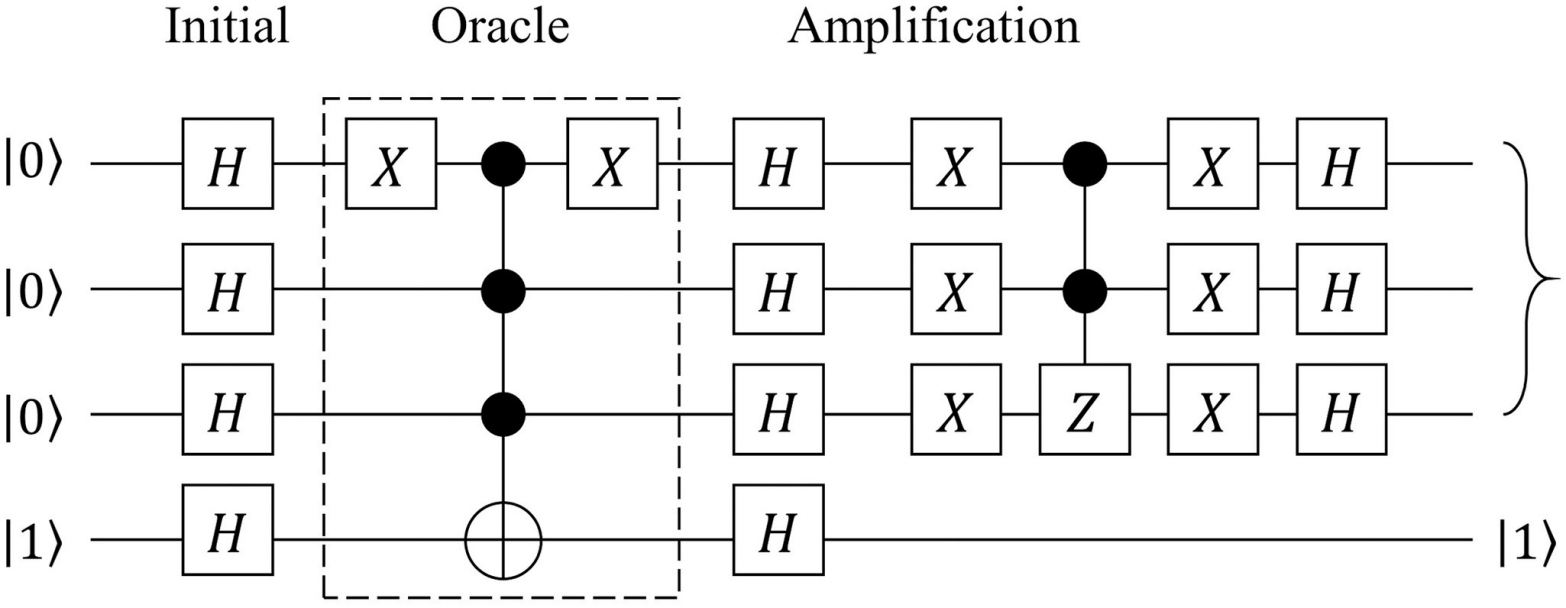
Quantum circuit of Grover’s algorithm. A quantum circuit implementing Grover’s algorithm with four qubits [50].

A *quantum circuit* is a diagram that represents the quantum algorithm as a sequence of quantum gates interconnected by qubit wires. Fig 1 shows an example of a graphical representation of a quantum circuit. The example presents a specific form of a 4-qubit quantum circuit of Grover’s algorithm [50], a well-known quantum algorithm for an unstructured search.

Each of the four horizontal lines represents the qubits, whereas the initial state of each qubit is represented as the starting point of each qubit wire. The objects located on the wires represent a quantum gate that performs a unitary transformation on the quantum states of the corresponding qubits. Some gates are located across multiple qubits with black circles on a few of the qubits. These are a type of controlled gate, and the black circles denote the control bits. After all gates are applied, the qubits are measured, and their quantum states collapse into one of the CBSs according to the measurement probability.

#### Boolean reversible functions

All operations in quantum computing are unitary operations, according to Definition 3. Among numerous unitary operations, *Boolean reversible functions* are frequently exploited as a key component in quantum algorithms [48]. For example, Grover’s algorithm in Fig 1 consists of three phases: initialization, oracle, and amplification. Among these phases, the *oracle phase* is a key part of the algorithm embedding the information about the target problem [51].

**Definition 5**. A *reversible function* is a one-to-one and bijective function of a finite set. In other words, the reversible function is a permutation. In particular, a *Boolean reversible function* is a multi-output reversible function composed of binary inputs and outputs.

*Example 3*. A Boolean reversible function is represented in several ways. Assume that a Boolean reversible function *f*: (*x*, *y*, *z*) → (*x*, *y*, *xy* ⊕ *z*) is given. Table 1(a) shows a matrix *P*_*f*_ representing the Boolean reversible function *f*. The matrix representing the Boolean reversible function appears as a permutation matrix. The same function *f* can also be represented as a truth table, as shown in Table 1(b).

**Table 1:** Representation of Boolean reversible function *f*.

(a) Matrix	(b) Truth Table							
$P_{f}=[\begin{array}{cccccccc} 1 & 0 & 0 & 0 & 0 & 0 & 0 & 0\\ 0 & 1 & 0 & 0 & 0 & 0 & 0 & 0\\ 0 & 0 & 1 & 0 & 0 & 0 & 0 & 0\\ 0 & 0 & 0 & 1 & 0 & 0 & 0 & 0\\ 0 & 0 & 0 & 0 & 1 & 0 & 0 & 0\\ 0 & 0 & 0 & 0 & 0 & 1 & 0 & 0\\ 0 & 0 & 0 & 0 & 0 & 0 & 0 & 1\\ 0 & 0 & 0 & 0 & 0 & 0 & 1 & 0 \end{array}]$	No.	Inputs			Outputs			Permutation
		*x*	*y*	*z*	*f*_1_	*f*_2_	*f*_3_	*F*
	0	0	0	0	0	0	0	0
	1	0	0	1	0	0	1	1
	2	0	1	0	0	1	1	2
	3	0	1	1	1	0	0	3
	4	1	0	0	1	0	0	4
	5	1	0	1	1	0	1	5
	6	1	1	0	1	1	1	7
	7	1	1	1	1	1	0	6

(a) Matrix form representation of the given Boolean reversible function *f*

(b) Truth table form representation of the given Boolean reversible function *f*

Boolean reversible functions can be classified into two types depending on the existence of the symbol “-” in the truth table: *completely specified function* and *incompletely specified function*.

**Definition 6**. We call the symbol “-” in the truth table an *unspecified bit*, which implies that the corresponding bit can be mapped either to 0 or 1. A *completely specified function* includes no unspecified bit on its truth table. Conversely, an *incompletely specified function* contains at least one unspecified bit in its truth table.

*Example 4*. Sample functions are shown in Table 2. Here, *peres* is a completely specified function, whereas *minialu* is an incompletely specified function. Note that if a given function is incompletely specified, the unspecified bits must be decided with either a 0 or 1 prior to the circuit implementation. At the same time, the output column must form a one-to-one correspondence with the input column because the given function is reversible.

**Table 2:** Truth tables of completely and incompletely specified functions.

(a) *peres*		(b) *minialu*			
Inputs	Outputs	Inputs	Outputs	Inputs	Outputs
0 0 0	0 0 0	0 0 0 0	- - 0 0	0 1 0 0	- - - 0
0 0 1	0 1 1	0 0 0 1	- - 0 1	0 1 0 1	- - - 1
0 1 0	0 1 0	0 0 1 0	- - 1 0	0 1 1 0	- - - 1
0 1 1	1 0 1	0 0 1 1	- - 1 1	0 1 1 1	- - - 1
1 0 0	1 0 0	1 0 0 0	- - - 0	1 1 0 0	- - 0 0
1 0 1	1 1 1	1 0 0 1	- - - 0	1 1 0 1	- - 0 1
1 1 0	1 1 0	1 0 1 0	- - - 0	1 1 1 0	- - 0 1
1 1 1	0 0 1	1 0 1 1	- - - 1	1 1 1 1	- - 1 0

(a) Completely specified function *peres*

(b) Incompletely specified function *minialu*

#### Reversible logic gates

Various types of gates, such as multiple control Toffoli gates, a multiple control Fredkin gate, and Peres gates, have been suggested to represent the Boolean reversible function in a circuit [14]. Among these types of gate libraries, a *multiple control Toffoli gate* is often introduced to express a Boolean reversible function.

**Definition 7**. A *multiple control Toffoli (MCT) gate* is a type of reversible logic gate that is composed of multiple control bits and a single target bit. The gate *C*^*m*^ *NOT*(*x*_1_, ⋯, *x*_*m*_;*x*_*m*+1_) implies an MCT gate with control bits on the first *m* lines and the target bit on the last *x*_*m*+1_ line. If all lines corresponding to control bits carry a state of 1, the line with the target bit flips the corresponding state of the qubit.

In a quantum circuit, a control bit is denoted as a black circle, whereas a target bit is denoted as a white circle. Note that for *m* = 0, 1, MCT gates are respectively equivalent to the Pauli *X* gate and the CNOT gate. When *m* = 2, the gate is called a Toffoli gate.

Most reversible logic gates cannot be implemented by single physical gates in a quantum computing environment. Thus, the reversible logic gate must be decomposed into a number of quantum basic gates. In the case of an MCT gate, as the number of control bits increases, the gate becomes more difficult to implement. Among several cost models for implementing a quantum circuit and a quantum gate, our study adopts a model called *quantum cost*.

**Fig 2:**
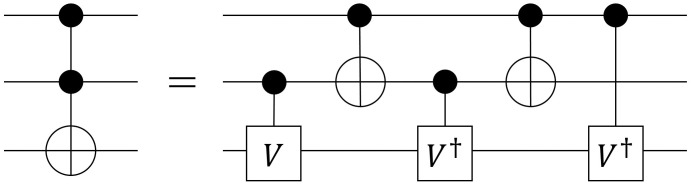
Toffoli gate implementation with basic quantum gates. A circuit composed of five basic quantum gates implementing the Toffoli gate *C*^2^ *NOT*(1, 2;3).

*Quantum cost* refers to the number of basic quantum gates required to implement the given function. For example, a Toffoli gate *C*^2^ *NOT*(1, 2;3) can be decomposed into five quantum basic gates, as shown in Fig 2. This implies that the quantum cost of a Toffoli gate is 5 [15]. As mentioned earlier, when the number of control bits increases, the MCT gate becomes more difficult to implement with a higher quantum cost. Table 3 shows an increasing quantum cost as the number of control lines increases [35].

**Table 3:** Quantum costs of multiple control Toffoli gates.

Control line	Quantum Cost
0	1
1	1
2	5
3	13
4	26, if at least 2 lines are empty
	29, otherwise
5	50, if at least 4 lines are empty
	80, if at least 1-3 lines are empty
	125, otherwise

This table cites the quantum cost table presented in [35].

#### Multi-commodity network flow model

A *multi-commodity network flow (MCNF) model* is a well-known model in network optimization. The following formulation represents the MCNF model in the node–arc form. The model we propose in this study is also based on the following formulation structure. A set *N* denotes the set of all nodes in *G*, whereas *A* denotes the set of all arcs. A set *K* implies the set of all commodities consisting of commodity *k* with source node *S* and terminal node *T*. A parameter $c_{ij}^{k}$ denotes the unit flow cost on an arc (*i*, *j*) and $x_{ij}^{k}$ with the flow on arc (*i*, *j*). A parameter $b_{i}^{k}$ represents the supply and demand of commodity *k* at node *i*. Let *g*_*ij*_ be the arc capacity on arc (*i*, *j*), whereas $u_{ij}^{k}$ denotes the flow capacity of commodity *k* on arc (*i*, *j*). Without a loss of generality, assume that each unit of each commodity consumes one unit of capacity from each arc upon which the commodity flows.

$$\begin{array}{cc} \text{minimize} & \sum_{k\in K}\sum_{(i,j)\in A}c_{ij}^{k}x_{ij}^{k} \end{array}$$ (5a)

$$\begin{array}{cccc} \text{subject}\;\text{to} & \sum_{(i,j)\in A}x_{ij}^{k}-\sum_{(j,i)\in A}x_{ji}^{k}=b_{i}^{k} & & \;\forall i\in N,\forall k\in K \end{array}$$ (5b)

$$\begin{array}{cccc} & \sum_{k\in K}x_{ij}^{k}\leq g_{ij} & & \;\forall (i,j)\in A \end{array}$$ (5c)

$$\begin{array}{cccc} & 0\leq x_{ij}^{k}\leq u_{ij}^{k} & & \;\forall (i,j)\in A,\forall k\in K \end{array}$$ (5d)

Eq (5a) represents the objective function that minimizes the total cost required to carry the flow from the origin to the appropriate destination. Eq (5b) is a set of constraints that assign the given supply and demand to each node *i* and commodity *k*. Eq (5c) is a set of *bundle constraints* that assign the upper bound to the total flow of each arc. The last constraint in Eq (5d) guarantees a non-negative value to the decision variables $x_{ij}^{k}$ for arc (*i*, *j*) and commodity *k* without exceeding the upper bound $u_{ij}^{k}$.

### Quantum reversible circuit synthesis (QRCS) problem

In this section, we define the *quantum reversible circuit synthesis (QRCS)* problem in detail and introduce the underlying MCNF structure with a few examples following the notation presented in Eqs (5a)–(5d).

#### Input and output of QRCS problem

The input of the QRCS problem is a Boolean reversible function given in a truth table form. The output of the QRCS problem is a circuit composed of MCT gates (including a NOT gate and a CNOT gate), which is a realized version of a Boolean reversible function given as an input of the problem. The given Boolean reversible function can be realized as various feasible circuits of different versions. In particular, the resulting circuit must have the minimum quantum cost among these feasible circuits. The length of the states determines the number of qubits in the circuit in the given truth table. Moreover, the maximum number of MCT gates of the circuit is given to the model, thus limiting the size of the resulting circuit.

#### MCNF Representation of QRCS problem

We formulate the model of the QRCS problem as an extended version of the MCNF model. The conventional MCNF model charges the unit flow cost per single arc. However, in the extended MCNF model that uniquely appears in the QRCS problem, the cost is given according to the selected subset of arcs.

**Fig 3:**
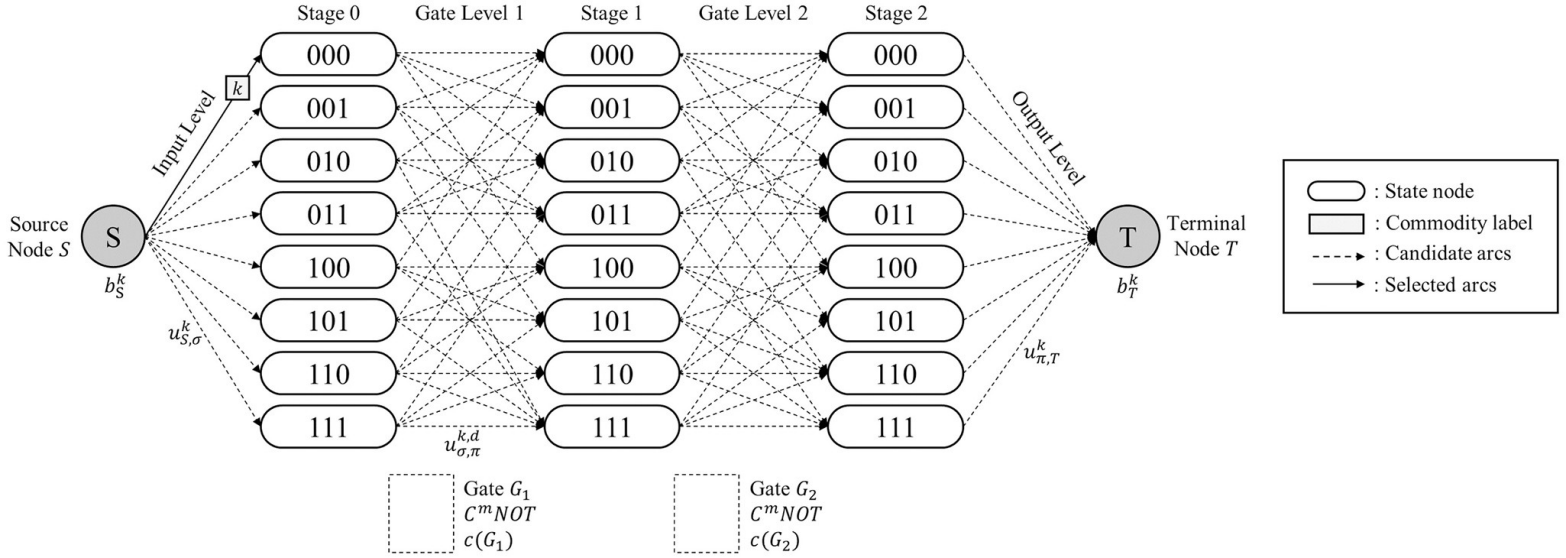
MCNF representation of the QRCS problem.

*Terminologies*. Suppose that a Boolean reversible function *F* is realized as a circuit composed of *N*_*Q*_ qubits and *N*_*D*_ MCT gates. Let a network G be a staged digraph composed of *N*_*D*_ + 1 successive *stages*. Each stage is composed of $2^{N_{Q}}$ *state nodes*, which is labeled by a *N*_*Q*_-bit binary string representing the corresponding CBS. All flows in the network G start from *source node* *S* and sink into *terminal node* *T*. Every state node in the initial stage is given an inflow of a specific commodity type from the source node *S*. Each commodity represents *N*_*K*_ elements in the output column of the given truth table. We call the conceptual space where the arcs are generated as *levels*. There are three types of levels depending on the components on both sides. An *input level* denotes the level between the source node *S* and the first stage. A *gate level* implies the level between two adjacent stages. An *output level* exists between the last stage and the terminal node *T*. Fig 3 shows the terminologies introduced in network G when *N*_*Q*_ = 3 and *N*_*D*_ = 2.

*Notations*. The notations used to represent the QRCS problem in the context of the MCNF model are presented in Fig 3. They follow the MCNF model presented in Eqs (5a)–(5d). The supply from the source node *S* of each commodity *k* is denoted as $b_{S}^{k}$, whereas the demand required at terminal node *T* of each commodity *k* is denoted as $b_{T}^{k}$. In the input level, the upper bound for each flow of the arc from source node *S* to the state node *σ* in stage 0 is denoted as $u_{S,\sigma }^{k}$ for commodity *k*. For arc (*σ*, *π*) in gate level *d*, the upper bound for each flow of the arc is denoted as $u_{\sigma ,\pi }^{k,d}$ for commodity *k*. In the output level, the upper bound for each flow of the arc (*S*, *π*) for a state node *π* in stage *N*_*D*_ is denoted as $u_{\pi ,T}^{k}$ for commodity *k*. Note that this demand parameter of each commodity enforces each flow carrying different commodities to arrive at the target state node in the final stage, resulting in an appropriate circuit realization of function *F*. Because all upper bounds of the flow are below 1, *g*_*ij*_ for every arc (*i*, *j*) in network G is set to 1. These parameters are preliminarily determined by function *F* given in a truth table form. However, the cost parameter of network G varies according to the selected network topology. In contrast to the original MCNF model, which includes a unit flow cost on each arc, the QRCS problem sets the network cost per gate assigned to each gate level. Therefore, instead of cost notation $c_{ij}^{k}$ for each arc (*i*, *j*) and commodity *k*, we denote the gate cost for gate *G*_*d*_ in gate level *d* as *c*(*G*_*d*_).

Some additional properties related to network topology are considered according to the characteristics of the QRCS problem. The properties are presented in the following three remarks.

**Remark 1**. An arc in network G can only be generated between two nodes in the adjacent stages. This implies that network G forms as a staged digraph.

**Remark 2**. An arc in the gate level of network G can only be generated when the Hamming distance between the two state nodes is less than or equal to 1. The remark is derived from the fact that each MCT gate includes only one target bit. Note that the Hamming distance implies the number of bit positions where the two bits contain different characters.

**Remark 3**. A set of arcs in a single gate level must form a bijective connection between the two groups of nodes in adjacent stages. This remark implies that the arcs in the gate level directly represent the permutation of CBS that occur by the assigned MCT gate.

#### Logic for MCT gate-network conversion

Each MCT gate *C*^*m*^ *NOT*(*x*_1_, ⋯, *x*_*m*_;*x*_*m*+1_) assigned to a single gate level is represented by a set of arcs denoting the permutation between the state nodes of two adjacent stages. The proposed model connotes the logic converting the assigned gate into a set of arcs in the network. Algorithm 1 shows the full logic as a pseudo code. The three conditions are nested to classify the cases into *CASE 1*, *CASE 2*, *CASE 3A* and *CASE 3B*.

**Algorithm 1**: MCT gate-network conversion

**for** *d*^*th*^ gate level for *d* ∈ {1, ⋯, *N*_*D*_} **do**

 Assign the gate *G*_*d*_ = *C*^*m*^ *NOT*(*x*_1_, ⋯, *x*_*m*_;*x*_*m*+1_)

 **if** no gate is assigned **then**

  *CASE 1* (Empty gate)

  **for** CBS *σ* in (*d* − 1)^*th*^ stage **do**

   Generate an arc (*σ*, *σ*) on *d*^*th*^ gate level

  **end**

 **else**

  **if** *m* = *0 for the gate* *G*_*d*_ **then**

   *CASE 2* (NOT gate)

   **for** CBS *σ* in (*d* − 1)^*th*^ stage **do**

    Compute $\tilde{\sigma }$ by performing the given NOT gate on *σ*

    Generate an arc $\left(\sigma ,\tilde{\sigma }\right)$ in *d*^*th*^ gate level

   **end**

  **else**

   *CASE 3* (MCT gate)

   **f**or CBS *σ* in (*d* − 1)^*th*^ stage **then**

    Conduct *sync test*

    **if** CBS *σ* has 1 in every digit *x*_1_, ⋯, *x*_*m*_ **then**

     *CASE 3A* (In sync with given MCT gate)

     Compute $\tilde{\sigma }$ by performing the given MCT gate on *σ*

     Generate an arc $\left(\sigma ,\tilde{\sigma }\right)$ in *d*^*th*^ gate level

    **else**

     *CASE 3B* (Not in sync with given MCT gate)

     Generate an arc (*σ*, *σ*) in *d*^*th*^ gate level

    **end**

   **end**

  **end**

 **end**

**end**

**Fig 4:**
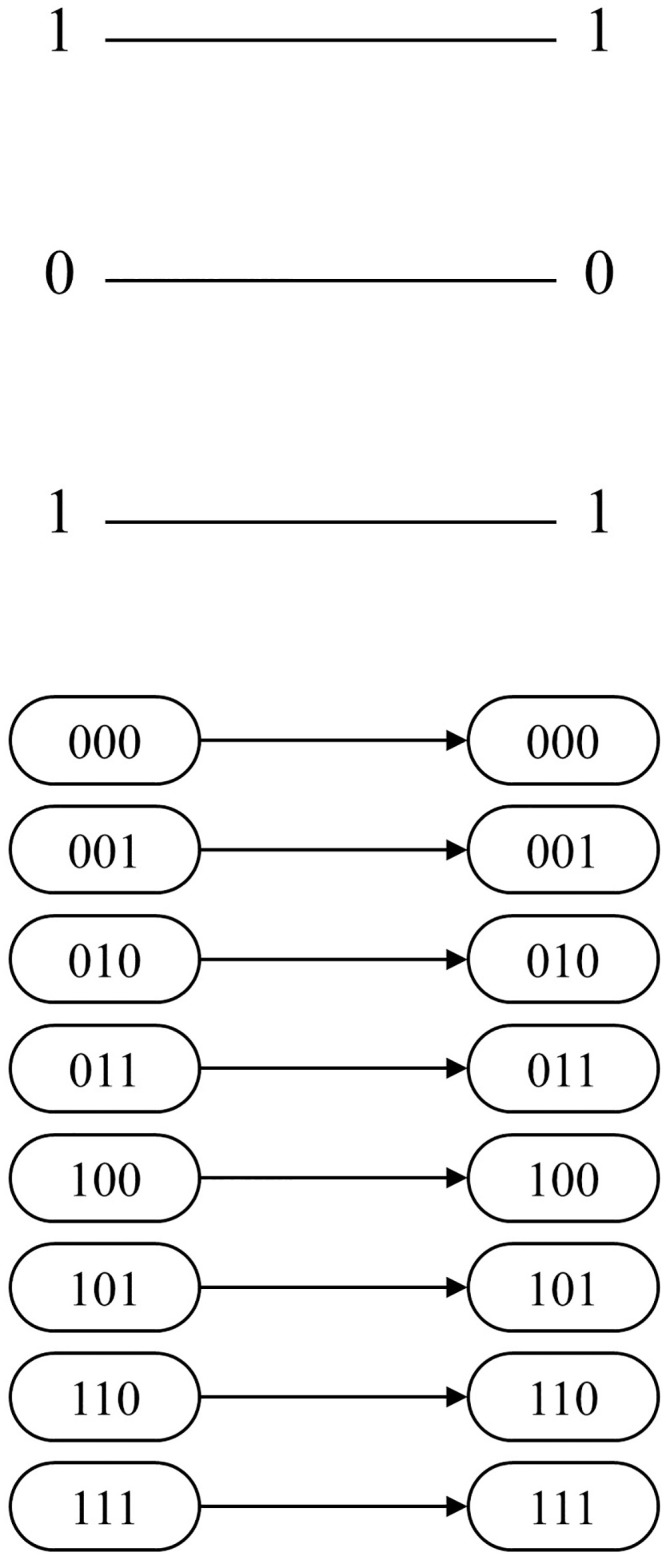
3-qubit example of MCT gate-network conversion: *CASE1*.

**Fig 5:**
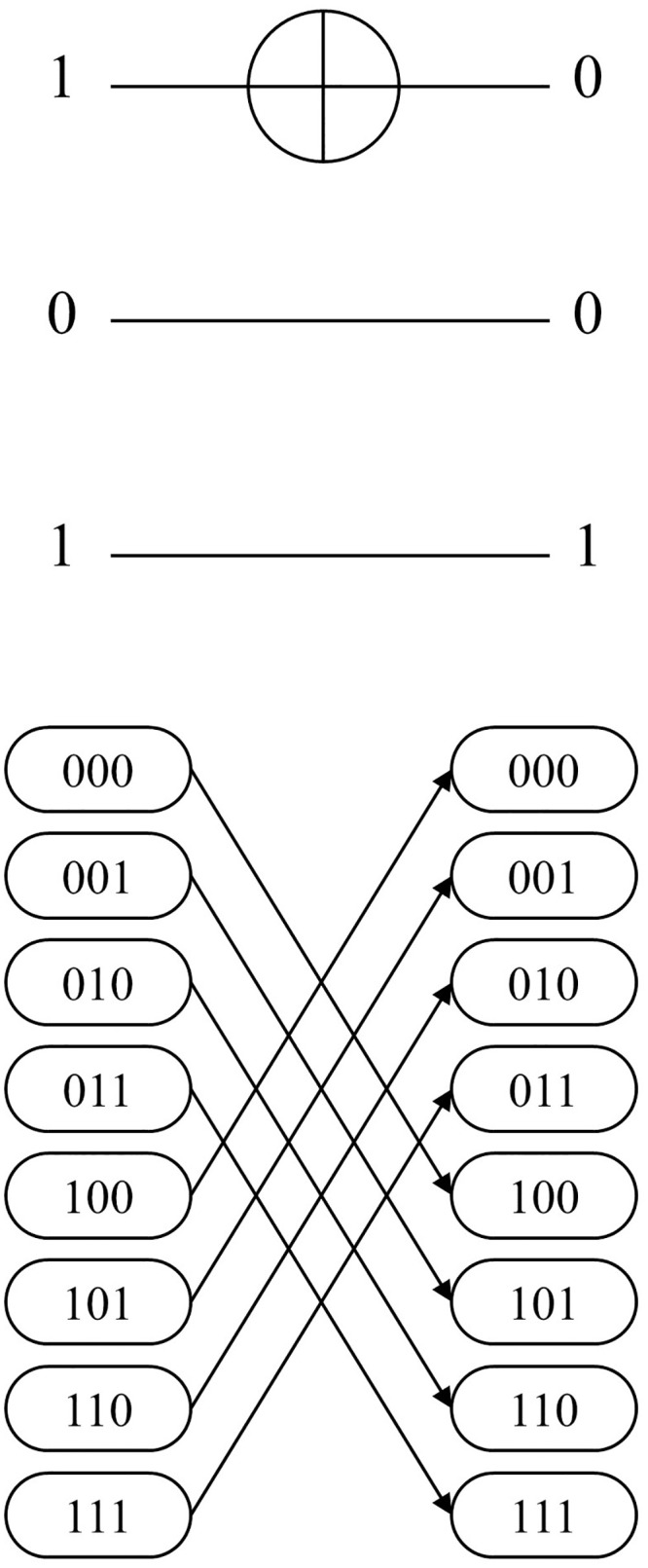
3-qubit example of MCT gate-network conversion: *CASE2*.

The first two conditions classify the cases in terms of the gate level. The first condition of Algorithm 1 checks whether a gate is assigned to the corresponding gate level. If no gate is assigned, the case is denoted as *CASE 1*. As shown in the network example of Fig 4, there are no permutations between two adjacent stages in the state nodes. Note that *CASE 1* occurs when the total number of MCT gates assigned in the circuit is less than the total number of gate levels given in the network layout. The second condition checks if the assigned MCT gate contains the control bits. If the given gate has no control bits, then the gate is considered to be a NOT gate. This case is denoted as *CASE 2*, and the corresponding network represents the permutation of the given NOT gate, as shown in Fig 5. The arcs (000, 100), (001, 101), (010, 110), and (011, 111) are formed because the first bit of each input CBS must be flipped. The last classification is for MCT gates, including at least one control bit. In contrast to the classification for *CASE 1* and *CASE 2*, the final classification is applied from the perspective of the CBS, following the rule referred to as *sync test*.

**Definition 8**. The *sync test* of gate *C*^*m*^ *NOT*(*x*_1_, ⋯, *x*_*m*_;*x*_*m*+1_) checks whether a CBS has the quantum state |1〉 in every control qubits *x*_1_, ⋯, *x*_*m*_. If the CBS passes the test, the CBS is *in sync* with the given MCT gate.

**Fig 6:**
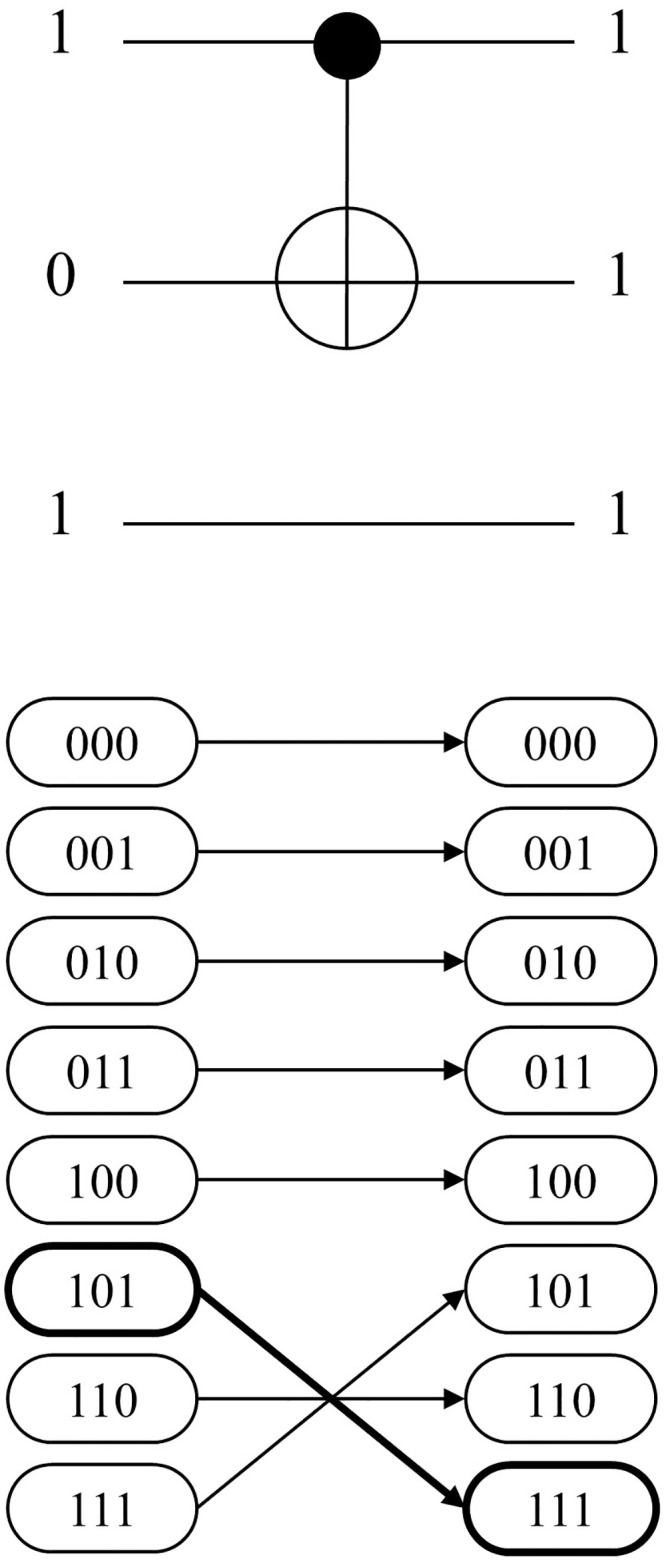
3-qubit example of MCT gate-network conversion: *CASE3A*.

**Fig 7:**
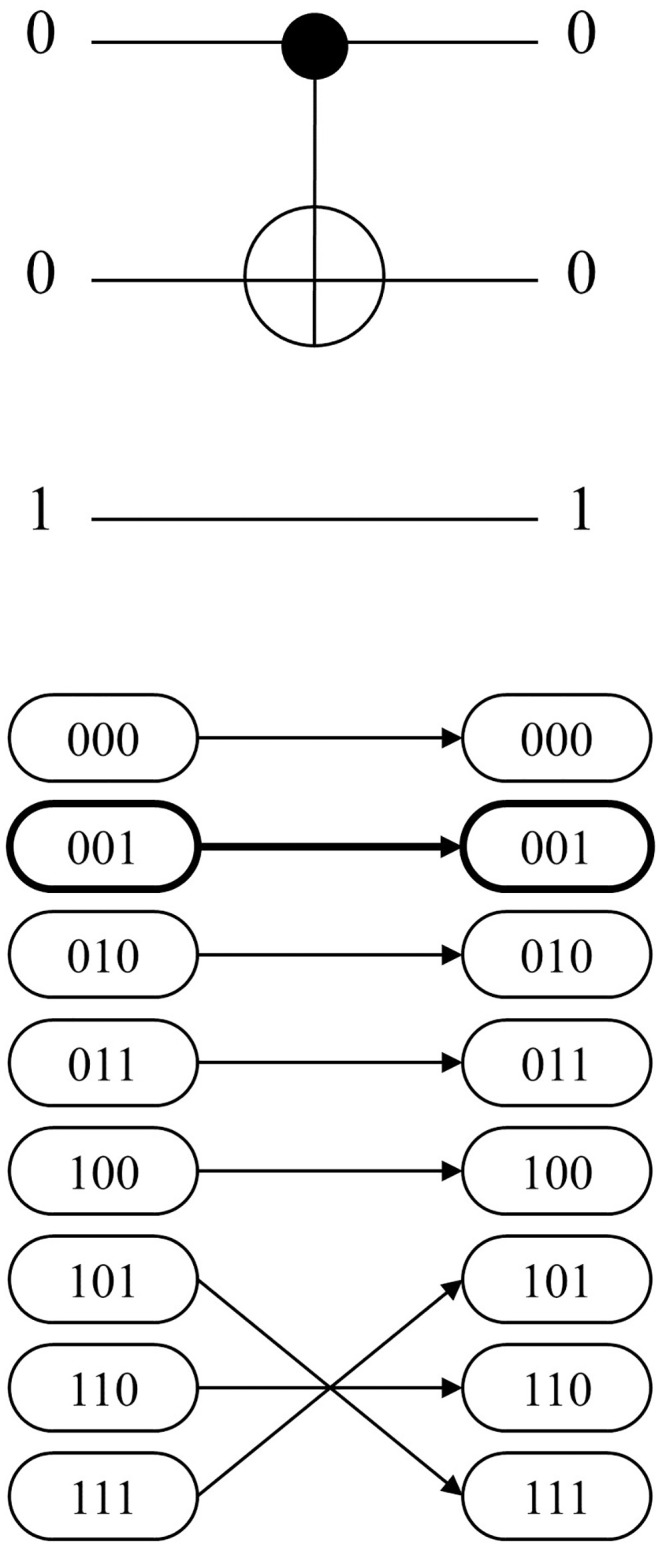
3-qubit example of MCT gate-network conversion: *CASE3B*.

Fig 6 shows an example of *CASE 3A*. The gate *C*^1^ *NOT*(1;2) and the input CBS 101 are given. The second bit of the CBS is flipped to 0 because the target bit is located on the second qubit. The network representation in Fig 6 also shows the corresponding change in state by forming arcs (101, 111) and (111, 101). Conversely, *CASE 3B* denotes the case in which the given CBS is not in sync with the assigned MCT gate. The example for CBS 001 is also given in Fig 7, which fails the sync test. Thus, the resulting state remains unchanged and the corresponding arc also makes no permutation. The logic in Algorithm 1 serves as the basic skeleton in developing the entire mathematical model, which is presented in the next section.

#### Descriptive examples

**Fig 8:**
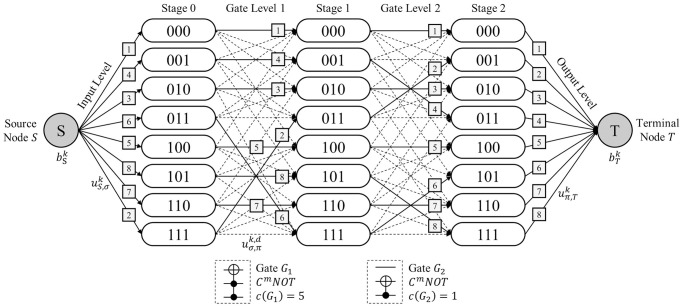
Network representation of MCT circuit for function *F*_1_.

*Example 5*. Fig 8 describes the MCNF representation of the QRCS problem when a completely specified function *F*_1_ with *N*_*Q*_ = 3 and *N*_*D*_ = 2 is given. Function *F*_1_ is given in Table 4 in a truth table form. Thus, the network is constructed using three stages, each composed of eight state nodes. Moreover, eight distinct types of commodities, *k* = 1, 2, ⋯, 8, originate from the source node *S* because function *F*_1_ is a completely specified function. The correspondence between the commodity index *k* and the output strings is presented in $b_{S}^{k},b_{T}^{k}$ in Table 4. Table 4 also includes the values of $u_{S,\sigma }^{k}$ and $u_{\pi ,T}^{k}$, which are determined according to the information given in the truth table of function *F*_1_. The dotted lines in Fig 8 denote the candidate arcs filtered based on Remark 2. The solid line implies a selected arc among these candidate arcs. Note that the arc selection follows the MCT gate-network conversion procedure of Algorithm 1 because *C*^2^ *NOT*(2, 3;1) gate *G*_1_ and *C*^1^ *NOT*(3;2) gate *G*_2_ are given. The total network cost, i.e., the quantum cost, is 6 because *c*(*G*_1_) = 5 and *c*(*G*_2_) = 1, according to Table 3. The commodity labels on the arcs of Fig 8 show that each flow is delivered to the appropriate destination through the selected arcs, while satisfying the upper bound constraints for each arc and the demand constraint of the terminal node.

**Table 4:** Truth table and parameters of completely specified function *F*_1_.

*F*_1_		$b_{S}^{k}$ , $b_{T}^{k}$				$u_{S,\sigma }^{k}$	*k*								$u_{\pi ,T}^{k}$	*k*							
Input	Output	*k*	Output	*S*	*T*	*σ*	1	2	3	4	5	6	7	8	*π*	1	2	3	4	5	6	7	8
000	000	1	000	1	-1	000	1	0	0	0	0	0	0	0	000	1	0	0	0	0	0	0	0
001	011	2	001	1	-1	001	0	0	0	1	0	0	0	0	001	0	1	0	0	0	0	0	0
010	010	3	010	1	-1	010	0	0	1	0	0	0	0	0	010	0	0	1	0	0	0	0	0
011	101	4	011	1	-1	011	0	0	0	0	0	1	0	0	011	0	0	0	1	0	0	0	0
100	100	5	100	1	-1	100	0	0	0	0	1	0	0	0	100	0	0	0	0	1	0	0	0
101	111	6	101	1	-1	101	0	0	0	0	0	0	0	1	101	0	0	0	0	0	1	0	0
110	110	7	110	1	-1	110	0	0	0	0	0	0	1	0	110	0	0	0	0	0	0	1	0
111	001	8	111	1	-1	111	0	1	0	0	0	0	0	0	111	0	0	0	0	0	0	0	1

**Fig 9:**
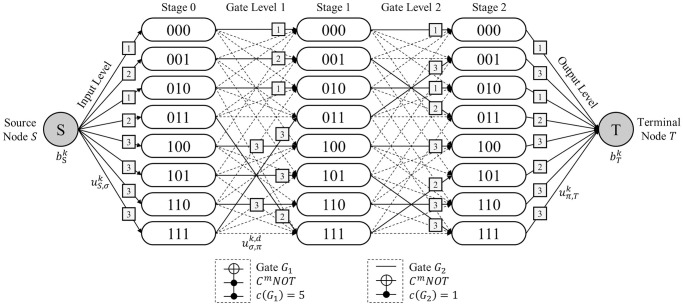
Network representation of MCT circuit for function *F*_2_.

**Example 6**. Fig 9 describes the MCNF representation of the QRCS problem when an incompletely specified function *F*_2_ with *N*_*Q*_ = 3 and *N*_*D*_ = 2 is given. Thus, the network is constructed using three stages, each composed of eight state nodes. The truth table of function *F*_2_ is given in Table 5. The function *F*_2_ is generated by masking a part of the bits in the output states of function *F*_1_ with the unspecified bits. The three types of states, i.e., - - 0, - - 1, and - - -, are the output column of the truth table. Therefore, three distinct types of commodities, *k* = 1, 2, 3, originate from the source node *S*. The correspondence between the commodity index *k* and the output strings is presented in the column $b_{S}^{k},b_{T}^{k}$ of Table 5. Table 5 also includes the values of $u_{S,\sigma }^{k}$ and $u_{\pi ,T}^{k}$ determined based on the given truth table of function *F*_2_. As shown in $u_{\pi ,T}^{k}$ of Table 5, the more varied way to send a flow to the terminal node *T* exists in the case of *F*_2_ because *F*_2_ is an incompletely specified function. However, note that the given feasible circuit is identical to Example 5; thus, the following result of the arc selection and network costs are the same. The commodity label on each of the arcs shows that all commodities are delivered to the appropriate destination while satisfying the upper bound constraints for each arc and the demand constraints of the terminal node.

**Table 5:** Truth table and parameters of incompletely specified function *F*_2_.

*F*_2_		$b_{S}^{k}$ , $b_{T}^{k}$				$u_{S,\sigma }^{k}$	*k*			$u_{\pi ,T}^{k}$	*k*		
Input	Output	*k*	Output	*S*	*T*	*σ*	1	2	3	*π*	1	2	3
000	- - 0	1	- - 0	2	-2	000	1	0	0	000	1	0	1
001	- - 1	2	- - 1	2	-2	001	0	1	0	001	0	1	1
010	- - 0	3	- - -	4	-4	010	1	0	0	010	1	0	1
011	- - 1					011	0	1	0	011	0	1	1
100	- - -					100	0	0	1	100	1	0	1
101	- - -					101	0	0	1	101	0	1	1
110	- - -					110	0	0	1	110	1	0	1
111	- - -					111	0	0	1	111	0	1	1

### Mathematical model

This section proposes the whole optimization model for solving the QRCS problem described in the preceding sections. The sets and parameters in the proposed model are listed in Table 6, whereas the decision variables and their corresponding definitions are listed in Table 7. The model imposes a total of 14 types of variables to construct the model. Moreover, a total of 30 types of constraints were developed to describe the QRCS problem.

$$\begin{array}{c} \text{minimize}Z=\sum_{d\in D}((\sum_{j\in Q\cup \left\{0\right\}-\left\{N_{Q}\right\}}c_{j}r_{j}^{d})-(1-\nu^{d})) \end{array}$$ (6a)

$$\begin{array}{cccc} \text{subject}\;\text{to} & \sum_{\left(\sigma ,\pi \right)\in H_{\sigma }^{1}}x_{\sigma ,\pi }^{k,1}=u_{S,\sigma }^{k} & & \forall \sigma \in \Omega ,\forall k\in K \end{array}$$ (6b)

$$\begin{array}{cccc} & \sum_{k\in K}\sum_{\pi \in C_{l}}x_{\pi ,l}^{k}=B_{T}^{l} & & \forall l\in K \end{array}$$ (6c)

$$\begin{array}{cccc} & \sum_{\left(\sigma ,\pi \right)\in H_{\sigma }^{1}}x_{\sigma ,\pi }^{k,d}-\sum_{\left(\pi ,\sigma \right)\in H_{\pi }^{1}}x_{\pi ,\sigma }^{k,d+1}=0 & & \forall k\in K,\forall \pi \in \Omega ,\forall d\in D/\{N_{D}\} \end{array}$$ (6d)

$$\begin{array}{cccc} & \sum_{\left(\sigma ,\pi \right)\in H_{\sigma }^{1}}x_{\sigma ,\pi }^{k,N_{D}}-\sum_{l\in C_{\pi }}x_{\pi ,l}^{k}=0 & & \forall k\in K,\forall \pi \in \Omega \end{array}$$ (6e)

$$\begin{array}{cccc} & \sum_{k\in K}x_{\sigma ,\pi }^{k,d}\leq 1 & & \forall \left(\sigma ,\pi \right)\in \underset{\sigma \in \Omega }{⋃}H_{\sigma }^{1},\forall d\in D \end{array}$$ (6f)

$$\begin{array}{cccc} & \sum_{k\in K}x_{\pi ,l}^{k,}\leq 1 & & \forall \left(\pi ,l\right)\in \underset{\pi \in \Omega }{⋃}\left\{\left(\pi ,l\right)|l\in C_{\pi }\right\} \end{array}$$ (6g)

$$\begin{array}{cccc} & \sum_{k\in K}x_{\sigma ,\pi }^{k,d}-\sum_{k\in K}x_{\pi ,\sigma }^{k,d}=0 & & \forall \left(\sigma ,\pi \right)\in \underset{\sigma \in \Omega }{⋃}H_{\sigma }^{1},\forall d\in D \end{array}$$ (6h)

$$\begin{array}{cccc} & t_{j}^{d}+w_{j}^{d}\leq 1 & & \forall d\in D,\forall j\in Q \end{array}$$ (6i)

$$\begin{array}{cccc} & \sum_{j\in Q}t_{j}^{d}-\nu^{d}=0 & & \forall d\in D \end{array}$$ (6j)

$$\begin{array}{cccc} & \nu^{d}-\nu^{d+1}\geq 0 & & \forall d\in D/\{N_{D}\} \end{array}$$ (6k)

$$\begin{array}{cccc} & {\bar{W}}^{d}-\sum_{j\in Q}w_{j}^{d}=0 & & \forall d\in D \end{array}$$ (6l)

$$\begin{array}{cccc} & {\bar{W}}^{d}-\sum_{j\in Q/\{N_{Q}\}}jr_{j}^{d}=0 & & \forall d\in D \end{array}$$ (6m)

$$\begin{array}{cccc} & \sum_{j\in Q/\{N_{Q}\}}r_{j}^{d}=1 & & \forall d\in D \end{array}$$ (6n)

$$\begin{array}{cccc} & u^{d}-{\bar{W}}^{d}\leq 0 & & \forall d\in D \end{array}$$ (6o)

$$\begin{array}{cccc} & {\bar{W}}^{d}-N_{Q}u^{d}\leq D & & \forall d\in D \end{array}$$ (6p)

$$\begin{array}{cccc} & \xi_{\sigma }^{d}-\sum_{j\in Q}w_{j}^{d}+\sum_{j\in Q}w_{j}^{d}\sigma_{j}\leq 0 & & \forall \sigma \in \Omega ,\forall d\in D \end{array}$$ (6q)

$$\begin{array}{cccc} & \sum_{j\in Q}w_{j}^{d}-\sum_{j\in Q}w_{j}^{d}\sigma_{j}-N_{Q}\xi_{\sigma }^{d}\leq 0 & & \forall \sigma \in \Omega ,\forall d\in D \end{array}$$ (6r)

$$\begin{array}{cccc} & \theta^{d}-\nu^{d}-h^{d}=1 & & \forall d\in D \end{array}$$ (6s)

$$\begin{array}{cccc} & \xi^{d}-u^{d}\leq 0 & & \forall d\in D \end{array}$$ (6t)

$$\begin{array}{cccc} & \sum_{k\in K}x_{\sigma ,\sigma }^{k,d}+\nu^{d}\geq 1 & & \forall \sigma \in \Omega ,\forall d\in D \end{array}$$ (6u)

$$\begin{array}{cccc} & t_{j}^{d}+h^{d}-\sum_{k\in K}x_{\sigma ,\sigma ⊕e_{j}}^{k,d}\leq 1 & & \forall \sigma \in \Omega ,\forall j\in Q,\forall d\in D \end{array}$$ (6v)

$$\begin{array}{cccc} & \sum_{k\in K}x_{\sigma ,\sigma ⊕e_{j}}^{k,d}-t_{j}^{d}+h^{d}\leq 1 & & \forall \sigma \in \Omega ,\forall j\in Q,\forall d\in D \end{array}$$ (6w)

$$\begin{array}{cccc} & h^{d}+\nu^{d}-u^{d}+3z^{d}\geq 2 & & \forall d\in D \end{array}$$ (6x)

$$\begin{array}{cccc} & h^{d}+\nu^{d}-u^{d}+2z^{d}\leq 2 & & \forall d\in D \end{array}$$ (6y)

$$\begin{array}{cccc} & \sum_{k\in K}x_{\sigma ,\sigma ⊕e_{j}}^{k,d}-f_{\sigma }^{d}\leq 0 & & \forall \sigma \in \Omega ,\forall j\in Q,\forall d\in D \end{array}$$ (6z)

$$\begin{array}{cccc} & \sum_{k\in K}x_{\sigma ,\sigma ⊕e_{j}}^{k,d}-t_{j}^{d}\leq 0 & & \forall \sigma \in \Omega ,\forall j\in Q,\forall d\in D \end{array}$$ (6aa)

$$\begin{array}{cccc} & f_{\sigma }^{d}+t_{j}^{d}-\sum_{k\in K}x_{\sigma ,\sigma ⊕e_{j}}^{k,d}\leq 1 & & \forall \sigma \in \Omega ,\forall j\in Q,\forall d\in D \end{array}$$ (6ab)

$$\begin{array}{cccc} & \sum_{k\in K}x_{\sigma ,\sigma }^{k,d}+f_{\sigma }^{d}\geq 1 & & \forall \sigma \in \Omega ,\forall j\in Q,\forall d\in D \end{array}$$ (6ac)

$$\begin{array}{cccc} & f_{\sigma }^{d}+u^{d}-\xi_{\sigma }^{d}+3y_{\sigma }^{d}+5\theta^{d}\geq 2 & & \forall \sigma \in \Omega ,\forall d\in D \end{array}$$ (6ad)

$$\begin{array}{cccc} & f_{\sigma }^{d}+u^{d}-\xi_{\sigma }^{d}+2y_{\sigma }^{d}-5\theta^{d}\leq 2 & & \forall \sigma \in \Omega ,\forall d\in D \end{array}$$ (6ae)

**Table 6:** Sets and parameters.

lX Notation	Definition
*N*_*Q*_	Number of qubits that compose the quantum circuit.
*N*_*D*_	Total number of gate levels that compose the network.
*N*_*K*_ ^(a)^	Number of commodity types in the network.
*D*	Set of indices for each gate level, *D* = {1, 2, ⋯, *N*_*D*_}.
*Q*	Set of indices for each qubit, *Q* = {1, 2, ⋯, *N*_*Q*_}.
*K*	Set of indices for each commodity type, *K* = {1, 2, ⋯, *N*_*K*_}.
Ω^(b)^	Set of indices for each state node, $\Omega =\{0_{\left(2\right)},1_{\left(2\right)},\cdots ,{\left(2^{N_{Q}}-1\right)}_{\left(2\right)}\}$.
$H_{\sigma }^{1}$	Set of paired indices for each candidate arc in gate levels with head node *σ* for *σ* ∈ Ω.
*S*	Source node of the network.
*T*	Terminal node of the network.
$u_{S,\sigma }^{k}$ ^(d)^	Upper bound of flow carrying commodity *k* on the arc from *S* to state node *σ*.
$u_{\pi ,T}^{k}$ ^(d)^	Upper bound of flow carrying commodity *k* on the arc from state node *π* to *T*.
$B_{T}^{l}$ ^(d)^	Total flow required in *T* carrying commodity *l* for *l* ∈ *K*, $B_{T}^{l}=-b_{T}^{l}$.
*C*_*π*_	Set of commodities that satisfy $u_{\pi ,T}^{k}=1$ for *π* ∈ Ω.
*C*_*l*_	Set of state nodes that satisfy $u_{\pi ,T}^{l}=1$ for *l* ∈ *K*.
*c*_*j*_	Quantum cost of an MCT gate containing *j* control bits for *j* ∈ *Q* ∪ {0}/{*N*_*Q*_}.
*σ*_*j*_	*j*^*th*^ bit in a binary string *σ* ∈ Ω.
*e*_*j*_ ^(d)^	Binary string of length *N*_*Q*_ with a 1 in *j*^*th*^ position, whereas the remaining bits are filled with zeros.

^(a)^ For a completely specified function, $N_{K}=2^{N_{Q}}$. Otherwise, $N_{K}<2^{N_{Q}}$.

^(b)^ For any non-negative integer $n<2^{N_{Q}}$, *n*_(2)_ implies a binary representation of integer *n* of fixed-length *N*_*Q*_. If the length is shorter than *N*_*Q*_, the remaining bits are filled with zeros from the first digit.

^(c)^ For example, if *N*_*Q*_ = 3, then *e*_3_ = 001.

^(d)^ The parameters are determined by the given Boolean reversible function.

**Table 7:** Decision variables.

lX Notation	Definition
$w_{j}^{d}$	A binary variable for *j* ∈ *Q*, *d* ∈ *D* that satisfies $w_{j}^{d}=1$ if a control bit is assigned to *j*^*th*^ qubit of the *d*^*th*^ gate in the circuit. Otherwise, $w_{j}^{d}=0$.
${\bar{W}}^{d}$	An integer variable for *d* ∈ *D* that indicates the number of control bits assigned to the *d*^*th*^ gate in the circuit.
$r_{j}^{d}$	A binary variable for *j* ∈ *Q*∪0 − {*N*_*Q*_}, *d* ∈ *D* that satisfies $r_{j}^{d}=1$ if a total of *j* control bits are assigned in the *d*^*th*^ gate. Otherwise, $r_{j}^{d}=0$.
$t_{j}^{d}$	A binary variable for *j* ∈ *Q*, *d* ∈ *D* that satisfies $t_{j}^{d}=1$ if a target bit is assigned to the *j*^*th*^ qubit of the *d*^*th*^ gate in the circuit. Otherwise, $t_{j}^{d}=0$.
$x_{\sigma ,\pi }^{k,d}$	A binary variable for $\left(\sigma ,\pi \right)\in \underset{\sigma \in \Omega }{⋃}H_{\sigma }^{\leq 1},k\in K,d\in D$ that satisfies $x_{\sigma ,\pi }^{k,d}=1$ if a candidate arc (*σ*, *π*) in the *d*^*th*^ gate level carries a flow of commodity type *k*. Otherwise, $x_{\sigma ,\pi }^{k,d}=0$.
$x_{\pi ,l}^{k}$	A binary variable for $\left(\pi ,l\right)\in \{\left(\pi ,l\right)|u_{\pi ,T}^{l}=1\}$, *k* ∈ *K* which satisfies $x_{\pi ,l}^{k}=1$ if a flow from *π* carrying commodity *k* is received as commodity *l* in node *T*. Otherwise, $x_{\pi ,l}^{k}=0$.
*ν*^*d*^ ^(a)^	A binary variable for *d* ∈ *D* that satisfies *ν*^*d*^ = 1 if a gate is assigned in the *d*^*th*^ gate level. Otherwise, *ν*^*d*^ = 0.
*u*^*d*^	A binary variable for *d* ∈ *D* that satisfies *u*_*d*_ = 1 if the gate in the *d*^*th*^ gate level contains more than one control bit. Otherwise, *u*_*d*_ = 0.
*h*^*d*^ ^(a)^	A binary variable ∀*d* ∈ *D* that satisfies *h*_*d*_ = 1 if the gate in the *d*^*th*^ gate level is a NOT gate. Otherwise, *h*_*d*_ = 0.
*z*^*d*^ ^(c)^	A binary variable for *d* ∈ *D* that composes an either-or constraint for *CASE 2*.
$\xi_{\sigma }^{d}$	A binary variable for *σ* ∈ Ω, *d* ∈ *D* that satisfies $\xi_{\sigma }^{d}=1$ if a CBS *σ* fails the sync test of the *d*^*th*^ MCT gate. Otherwise, $\xi_{\sigma }^{d}=0$.
*θ*^*d*^ ^(a)^	A binary variable for *d* ∈ *D* that satisfies *θ*^*d*^ = 0 if the gate in the *d*^*th*^ gate level is a *C*^*m*^ *NOT* gate for *m* ≥ 1. Otherwise, *θ*^*d*^ = 1.
$f_{\sigma }^{d}$ ^(b)^	A binary variable for *σ* ∈ Ω, *d* ∈ *D* that satisfies $f_{\sigma }^{d}=1$ if a CBS *σ* passes a sync test of the *d*^*th*^ MCT gate containing at least one control bit. Otherwise, $f_{\sigma }^{d}=0$.
$y_{\sigma }^{d}$ ^(d)^	A binary variable for *σ* ∈ Ω, *d* ∈ *D* that composes an either-or constraint for *CASE 3A* and *CASE 3A*.

^(a)^ *ν*_*d*_, *h*_*d*_, and *θ*_*d*_ are the discriminators for *CASE 1*, *CASE 2*, and *CASE 3*, repectively.

^(b)^ $f_{\sigma }^{d}$ is a discriminator for *CASE 3A* ($f_{\sigma }^{d}=1$) and *CASE 3B* ($f_{\sigma }^{d}=0$).

^(c)^ *z*_*d*_ works jointly with *h*^*d*^ to classify *CASE 2* through the values of *ν*^*d*^ and *u*^*d*^.

^(d)^ $y_{\sigma }^{d}$ works jointly with $f_{\sigma }^{d}$ to classify *CASE 3A* and *CASE 3B* through the values of *u*^*d*^ and $\xi_{\sigma }^{d}$.

Eq (6a) is an objective function of the model that minimizes a sum of the quantum cost of the decided MCT circuit. From a network optimization perspective, the objective function implies minimizing the total network cost, which is charged according to the subset of arcs in each gate level. Eqs (6b)–(6h) are the constraints that determine the network structure. In particular, Eqs (6b)–(6g) are constraints based on the MCNF model. In addition, Eqs (6b) and (6c) define the commodity constraint respectively on the initial stage and output level. Eq (6b) defines the initial input commodity of each inflow from the source node. The initial flows are already decided as parameters according to the target output in the given truth table. Eq (6c) defines the total demand of inflow for each commodity received at the terminal node. Eqs (6d) and (6e) are the conservation constraints of the flow for the nodes in the stages. In addition, Eqs (6f) and (6g) are the bundle constraints for the arcs in the gate and output levels, respectively. Because the network represents the permutation, Eq (6h) forces the symmetry in the arcs of each gate level.

Eqs (6i)–(6p) are constraints related to the generation of the circuit and gates. Eq (6i) implies that the control and target bits cannot be assigned to the same location of the circuit. By Eq (6j), if an MCT gate is assigned to a gate level, the gate must include a target bit. Eq (6k) assigns the MCT gate on the consecutive gate levels because, in some cases, not all levels are filled with gates. Eqs (6l)–(6n) sum the number of control bits for each gate. Eqs (6o) and (6p) suppress the assignment of control bits to the circuit if a gate is not assigned to the corresponding level.

Eqs (6q)–(6t) are preliminary constraints for the gate classification and the sync test. In addition, Eqs (6q) and (6r) express the sync test of the MCT gate, Eq (6s) defines the auxiliary variable for *CASE 3*, and Eq (6t) allows the CBS to pass the sync test when the corresponding gate does not contain the control bit.

Eqs (6u)–(6ac) are constraints that embody the MCT gate-network conversion described in Algorithm 1. Eq (6u) determines the arcs at the gate level when the gate is not assigned, which is denoted as *CASE 1*. In this case, every state node forms an arc with the state node that has an identical node label. Eqs (6v) and (6w) work jointly to determine the arcs of the gate level when a NOT gate is assigned, which is denoted as *CASE 2*. Eqs (6x) and (6y) are either-or constraints that discriminate *CASE 2* with two types of variables, i.e., *ν*^*d*^, indicating that a gate is assigned to a gate level, and *u*^*d*^, indicating that a control bit is contained in the assigned gate. Eqs (6z)–(6ab) are the constraints determining the arcs of the gate level in *CASE 3A*. Conversely, Eq (6ac) is a constraint that determines the arcs of the gate level in *CASE 3B*. Eqs (6ab) and (6ae) are the either-or constraints that discriminate *CASE 3A* and *CASE 3B* using two types of variables, i.e., *u*^*d*^, indicating if an assigned gate contains the control bit, and $\xi_{\sigma }^{d}$, indicating if a CBS passes the sync test of the gate.

## Results

In this section, we provide details of the computational experiments on the proposed optimization model. The optimization model proposed in the previous section was implemented and compiled using Python 3.6.6. The experiment was conducted using Windows 10 OS on a personal computing with a 3.20-GHz Intel Core^™^ i7-8700U CPU with 16.00 GB of memory. The problems were solved by using Gurobi 9.0.0, and the maximum calculation time was set to 36,000s. If the optimal solution was not found within the maximum calculation time, the best feasible solution found was printed. All other parameters were set to their default values. The computational experiments on the proposed model used 44 Boolean reversible functions obtained from revlib [52]. Among the benchmark data, including up to 6 qubits, we used data commonly applied in previous studies.

The computational experiments were conducted by varying the values of *N*_*D*_ from 1 to 8. Table 8 compares the computational result of *N*_*D*_ = 7 with the results of prior studies in terms of quantum costs. In the last column of Table 8, Δ, the percentage of change in quantum cost for the solution to the case of *N*_*D*_ = 7 by the proposed model is compared to the best solution among the previous studies. The positive value in column Δ column implies that the proposed model derives a solution with an improved quantum cost compared to the best results among the previous studies.

**Table 8:** Comparison of computational results with those of previous studies.

No.	Function	C/I	*N*_*Q*_	Previous studies										Best among previous studies		Proposed model *N*_*D*_ = 7		
				[53]		[54]		[55]		[52]		[38]						
				Gate	QC	Gate	QC	Gate	QC	Gate	QC	Gate	QC	Gate	QC	Gate	QC	Δ
1	fredkin	C	3			3	15							3	15	3	7	53.3
2	peres	C	3			2	6							2	6	2	6	0.0
3	ham3	C	3							5	9	5	9	5	9	5	9	0.0
4	miller	C	3			5	17							5	17	5	9	47.1
5	3_17	C	3			6	14					6	14	6	14	6	14	0.0
6	ex_1	C	3							4	8	4	8	4	8	4	8	0.0
7	toffoli_double	C	4							2	10			2	10	3	7	30.0
8	mod5d1	C	5			7	11					7	11	7	11	7	11	0.0
9	mod5d2	C	5	8	16	8	20							8	16	-	-	- ^(a)^
10	mod5mils	C	5	5	13	5	13					5	13	5	13	6	10	23.1
11	graycode6	C	6									5	5	5	5	5	5	0.0
12	decod24_v0	I	4			6	18					6	18	6	18	6	10	44.4
13	decod24_v1	I	4			6	22							6	22	7	11	50.0
14	decod24_v2	I	4			6	18							6	18	7	11	38.9
15	decod24_v3	I	4			7	35							7	35	7	11	68.6
16	rd32_v0	I	4			4	12					4	12	4	12	5	9	25.0
17	rd32_v1	I	4			5	13							5	13	6	10	23.1
18	mini_alu	I	4			5	33			6	62			5	33	7	19	42.4
19	mod10	I	4							7	43			7	43	7	27	37.2
20	alu_v0	I	5			6	22	6	14			6	22	6	14	6	14	0.0
21	alu_v1	I	5			7	15	7	15					7	15	7	15	0.0
22	alu_v2	I	5	13	101	7	39	7	15					7	15	7	15	0.0
23	alu_v3	I	5			7	19	7	15	7					15	7	15	0.0
24	alu_v4	I	5			7	31	7	15					7	15	7	15	0.0
25	4gt4_v0	I	5	17	89	6	54							6	54	7	19	64.8
26	4gt4_v1	I	5			5	57							5	57	7	19	66.7
27	4gt5_v0	I	5	13	29	5	21							5	21	5	13	38.1
28	4gt5_v1	I	5			4	28					4	16	4	16	5	13	18.8
29	4gt10_v0	I	5	15	53	5	37			9	49			5	37	6	18	51.4
30	4gt10_v1	I	5			6	34							6	34	7	19	44.1
31	4gt11_v0	I	5	12	16	3	7			8	12	3	7	3	7	3	7	0.0
32	4gt11_v1	I	5			4	8							4	8	4	8	0.0
33	4gt12_v0	I	5	14	58	5	41			10	54	5	37	5	37	6	22	40.5
34	4gt12_v1	I	5			5	45							5	45	7	23	48.9
35	4gt13_v0	I	5	14	34	3	15			10	30	3	15	3	15	3	15	0.0
36	4gt13_v1	I	5			4	16							4	16	4	16	0.0
37	4mod7_v0	I	5	6	38	6	38							6	38	7	39	-2.6
38	4mod7_v1	I	5			5	39							5	39	7	39	0.0
39	one_two_three_v0	I	5	11	71	8	40							8	40	8 ^(b)^	20	50.0
40	one_two_three_v1	I	5			8	36							8	36	8 ^(b)^	24	33.3
41	one_two_three_v2	I	5			8	24							8	24	8 ^(b)^	16	33.3
42	one_two_three_v3	I	5			8	24							8	24	8 ^(b)^	24	0.0
43	4mod5_v0	I	5			5	9					5	9	5	9	5	9	0.0
44	4mod5_v1	I	5			5	13	4	7					4	7	5	9	-28.6

C/I: C for a completely specified function, I for an incompletely specified function

Gate: Number of MCT gates composing the circuit/QC: Quantum cost of the circuit

Δ(%): Percentage of improvement in the result of the proposed model in terms of quantum costs, compared to the best solution among previous studies

^(a)^ A feasible solution to data No. 9 under *N*_*D*_ = 7 was not found within the time limit.

^(b)^ The solution under *N*_*D*_ = 8 is presented as at least eight MCT gates being required to implement function Nos.39–42, *one_two_three*.

For 23 cases out of a total of 44 functions, the circuit showed an improvement in terms of the quantum costs. The improvement ratio was a minimum 18.8% to a maximum of 68.6%. For 18 cases, our model obtained the same result as the previous studies regarding the quantum costs. Some cases showed an optimality gap of 0.0%, which indicates that the obtained solution is optimal in terms of the quantum costs. This implies that the proposed model can evaluate the computational performance of the proposed circuit synthesis heuristics. Case No. 9 *mod5d2* failed to find a feasible solution within the time limit from the remaining three cases. The other two cases showed a higher quantum cost than the previous results. For No. 37, *4mod7_v0*, the optimality gap appeared to be 84.6% even with the maximum computation time. Thus, we expect that a longer computation time is required to reach the same or better computational result than in previous studies. However, in case No. 44, *4mod5_v1*, we derived the optimal circuit of quantum cost 9, which is composed of five MCT gates from the proposed model. Thus, we carefully presume that there appears to be an error in the previous result. Overall, the computational result shows that the proposed methodology significantly improves the quantum costs.

Table 9 shows the full computational result of the proposed model. As *N*_*D*_ and *N*_*Q*_ increase, more nodes are explored when searching for the feasible or optimal solution. Accordingly, the required computational time also increases. As noted in Table 9, the total quantum cost tends to decrease as *N*_*D*_ increases in many cases. However, in some cases with *N*_*D*_ = 8, the optimality gap is insufficiently reduced within the time limit, resulting in a solution with a slightly higher quantum cost than in the case of *N*_*D*_ = 7. These cases have lower quantum costs than even the cases = of *N*_*D*_ ≤ 5.

**Table 9:** Computational results with different *N*_*D*_.

Index	Function name	C/I	*N*_*Q*_	Best solution among *N*_*D*_ ≤ 5						*N*_*D*_ = 6					*N*_*D*_ = 7					*N*_*D*_ = 8				
				*N*_*D*_	Gate	QC	Gap	Time	Node	Gate	QC	Gap	Time	Node	Gate	QC	Gap	Time	Node	Gate	QC	Gap	Time	Node
1	fredkin	C	3	5	3	7	0.0	1	341	3	7	0.0	19	7055	3	7	0.0	5	2897	3	7	0.0	4	1347
2	peres	C	3	5	2	6	0.0	1	729	2	6	0.0	2	1456	2	6	0.0	2	821	2	6	0.0	3	1054
3	ham3	C	3	5	5	9	0.0	1	88	5	9	0.0	6	959	5	9	0.0	33	8174	5	9	0.0	306	47602
4	miller	C	3	5	5	9	0.0	1	78	5	9	0.0	3	581	5	9	0.0	27	7451	5	9	0.0	245	39168
5	3_17	C	3	-	-	-	-	2	81	6	14	0.0	5	947	6	14	0.0	22	6737	6	14	0.0	173	42601
6	ex_1	C	3	5	4	8	0.0	1	264	4	8	0.0	4	1163	4	8	0.0	23	9538	4	8	0.0	15	11390
7	toffoli_double	C	4	5	3	7	0.0	36	2605	3	7	0.0	608	18934	3	7	0.0	182	3984	3	7	0.0	191	1640
8	mod5d1	C	5	-	-	-	-	500	2849	-	-	-	33586	24813	7	11	54.5	36000	7859	8	24	83.3	36000	2488
9	mod5d2	C	5	-	-	-	-	65	1	-	-	-	9949	975	-	-	-	36000	3242	-	-	-	36000	1155
10	mod5mils	C	5	5	5	13	0.0	729	1406	6	10	40.0	36000	7381	6	10	50.0	36000	3511	6	10	50.0	36000	1666
11	graycode6	C	6	5	5	5	0.0	9	0	5	5	0.0	2145	1	5	5	0.0	5144	1	8	8	37.5	36000	1
12	decod24_v0	I	4	-	-	-	-	5	498	6	10	0.0	34	5449	6	10	0.0	185	13729	6	10	0.0	2342	247649
13	decod24_v1	I	4	-	-	-	-	7	650	6	14	0.0	36	7912	7	11	0.0	244	16908	7	11	0.0	1402	88010
14	decod24_v2	I	4	-	-	-	-	6	503	6	14	0.0	88	11253	7	11	0.0	172	10429	7	11	0.0	2079	162105
15	decod24_v3	I	4	-	-	-	-	8	798	-	-	-	34	7059	7	11	0.0	358	19005	7	11	0.0	1426	66886
16	rd32_v0	I	4	5	5	9	0.0	19	3846	5	9	0.0	170	11443	5	9	0.0	3997	484100	5	9	11.1	36000	3832869
17	rd32_v1	I	4	5	5	13	0.0	13	2666	6	10	0.0	167	23580	6	10	0.0	3191	405732	6	10	20.0	36001	3700460
18	mini_alu	I	4	5	5	25	0.0	23	2083	6	22	0.0	237	25844	7	19	0.0	7529	454341	7	19	57.9	36000	1452366
19	mod10	I	4	-	-	-	-	17	342	6	30	0.0	142	4442	7	27	0.0	1087	14040	8	20	0.0	11725	120732
20	alu_v0	I	5	-	-	-	-	42	5207	6	14	0.0	780	36819	6	14	0.0	17963	527448	7	15	60.0	36000	510597
21	alu_v1	I	5	-	-	-	-	294	6076	-	-	-	1163	21574	7	15	0.0	20503	269789	8	16	62.5	36000	313732
22	alu_v2	I	5	-	-	-	-	199	5431	-	-	-	3923	57282	7	15	0.0	32147	595676	7	15	60.0	36000	694169
23	alu_v3	I	5	-	-	-	-	57	2189	-	-	-	1362	37397	7	15	0.0	9009	252350	7	15	53.3	36000	885082
24	alu_v4	I	5	-	-	-	-	29	1870	-	-	-	1598	36909	7	15	0.0	11354	394906	7	15	53.3	36000	849367
25	4gt4_v0	I	5	-	-	-	-	152	8895	6	22	0.0	13315	403536	7	19	73.7	36000	754981	8	20	75.0	36000	806978
26	4gt4_v1	I	5	5	5	29	0.0	310	11221	6	18	0.0	33574	1043472	7	19	73.7	36000	1016556	7	19	73.7	36000	739778
27	4gt5_v0	I	5	5	5	13	0.0	350	11327	5	13	0.0	11157	275685	5	13	53.9	36000	1144365	5	13	53.9	36000	858094
28	4gt5_v1	I	5	5	5	13	0.0	259	8806	5	13	0.0	8813	322085	5	13	53.9	36000	1229372	5	13	53.9	36000	939766
29	4gt10_v0	I	5		5	21	0.0	283	12032	6	18	0.0	20601	694808	6	18	72.2	36000	892034	8	20	75.0	36000	734223
30	4gt10_v1	I	5	-	-	-	-	171	8600	6	18	0.0	19275	518172	7	19	73.7	36000	1074299	8	20	75.0	36000	595028
31	4gt11_v0	I	5	5	3	7	0.0	130	7228	3	7	0.0	3452	72923	3	7	0.0	1208	28075	3	7	0.0	834	15140
32	4gt11_v1	I	5	5	4	8	0.0	91	11281	4	8	0.0	1034	29472	4	8	12.5	36000	1471249	4	8	12.5	36000	761025
33	4gt12_v0	I	5	5	5	25	0.0	295	19958	6	22	0.0	11605	340106	6	22	77.3	36000	846115	6	22	77.3	36000	675819
34	4gt12_v1	I	5	5	5	45	0.0	347	9637	6	26	0.0	7437	226915	7	23	73.9	36000	773936	7	23	78.3	36000	418497
35	4gt13_v0	I	5	5	3	15	0.0	250	7955	3	15	0.0	7731	233449	3	15	60.0	36000	1042868	3	15	66.7	36000	591244
36	4gt13_v1	I	5	5	4	16	0.0	183	10350	4	16	0.0	7380	192324	4	16	62.5	36000	788703	4	16	68.8	36000	540699
37	4mod7_v0	I	5	-	-	-	-	277	1515	-	-	-	3273	20345	7	39	84.6	36000	105314	8	32	81.3	36000	77169
38	4mod7_v1	I	5	-	-	-	-	137	920	-	-	-	3273	20345	7	39	84.6	36000	105314	8	32	81.3	36000	77169
39	one_two_three_v0	I	5	-	-	-	-	80	1311	-	-	-	1080	14005	-	-	-	9586	141265	8	20	70.0	36000	278736
40	one_two_three_v1	I	5	-	-	-	-	45	902	-	-	-	1255	27743	-	-	-	13662	190104	8	24	75.0	36000	264527
41	one_two_three_v2	I	5	-	-	-	-	55	1064	-	-	-	1141	15947	-	-	-	10595	112521	8	16	62.5	36000	266788
42	one_two_three_v3	I	5	-	-	-	-	62	1368	-	-	-	370	8783	-	-	-	6661	102340	8	24	70.8	36000	424232
43	4mod5_v0	I	5	5	5	9	0.0	132	8783	5	9	0.0	2277	53674	5	9	22.2	36000	947237	5	9	33.3	36000	1366449
44	4mod5_v1	I	5	5	5	9	0.0	109	6668	5	9	0.0	1264	33526	5	9	33.3	36000	1190263	5	9	33.3	36000	1080344

Gap: Optimality gap in percentage / Time: Computational time in seconds / Node: Number of explored node in branching tree / -: Infeasible cases or time limit reached

**Fig 10:**
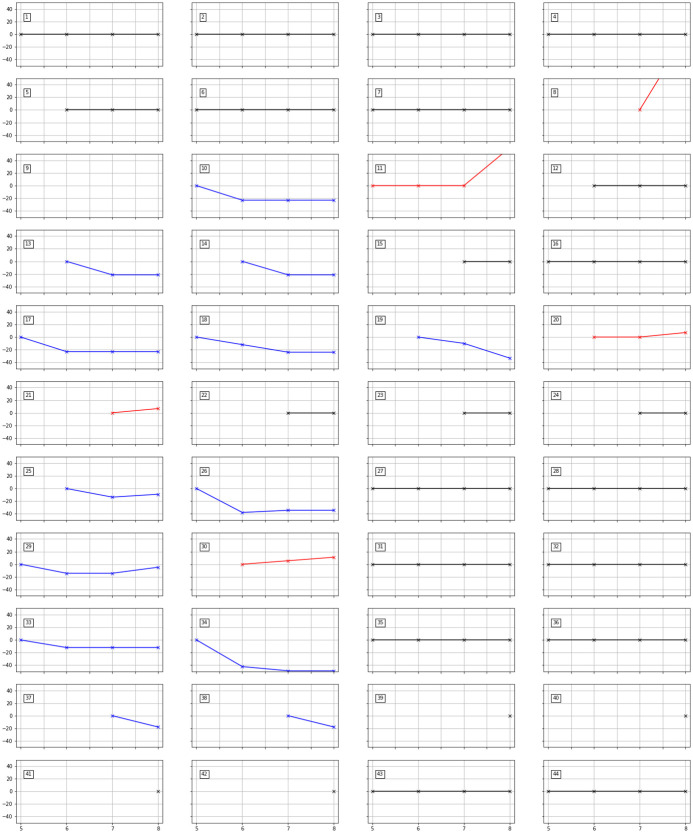
Change in quantum cost as *N*_*D*_ increases. The number on the top-left of each plot indicates the data index in Table 9.

Fig 10 visualizes the tendency of quantum costs varying with *N*_*D*_. Each plot shows the change in quantum costs as *N*_*D*_ increases from 5 to 8. The vertical axis indicates the normalized value of quantum costs compared to the best solution among *N*_*D*_ ≤ 5. The number on the top-left of each plot indicates the data index from Table 9. The plots with black lines imply that no change in quantum costs occurred in the corresponding case. Conversely, the plot with blue lines shows the decrease in the quantum costs as *N*_*D*_ increases. However, note that among some of these cases, the quantum cost slightly increases in *N*_*D*_ = 8 cases because the optimality gap is not sufficiently narrowed within the limited computational time.

**Fig 11:**
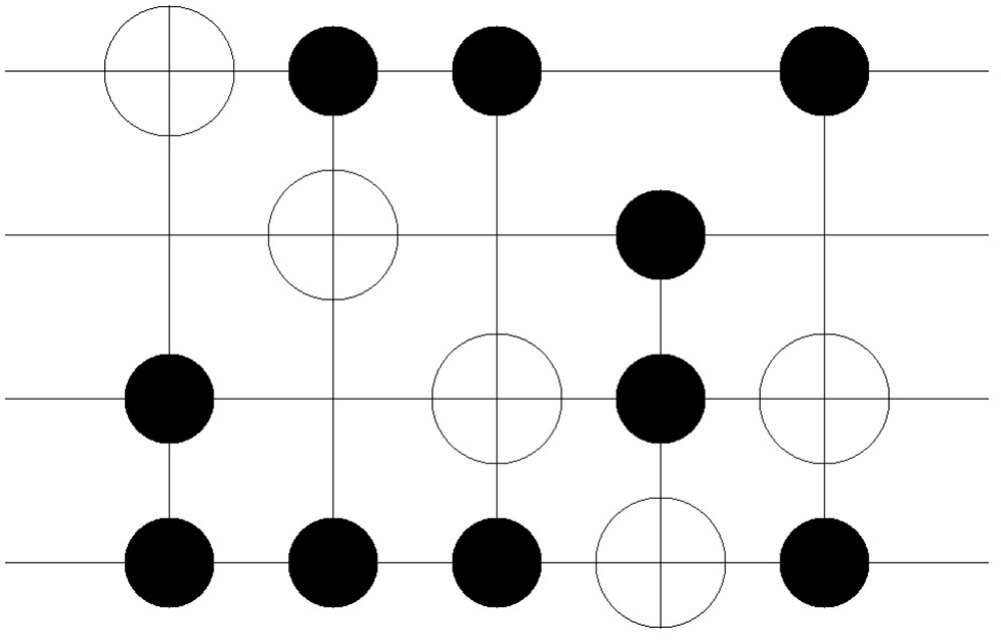
Resulting circuits of No. 18 *mini_alu* with varying *N*_*D*_: *N*_*D*_ = 5.

**Fig 12:**
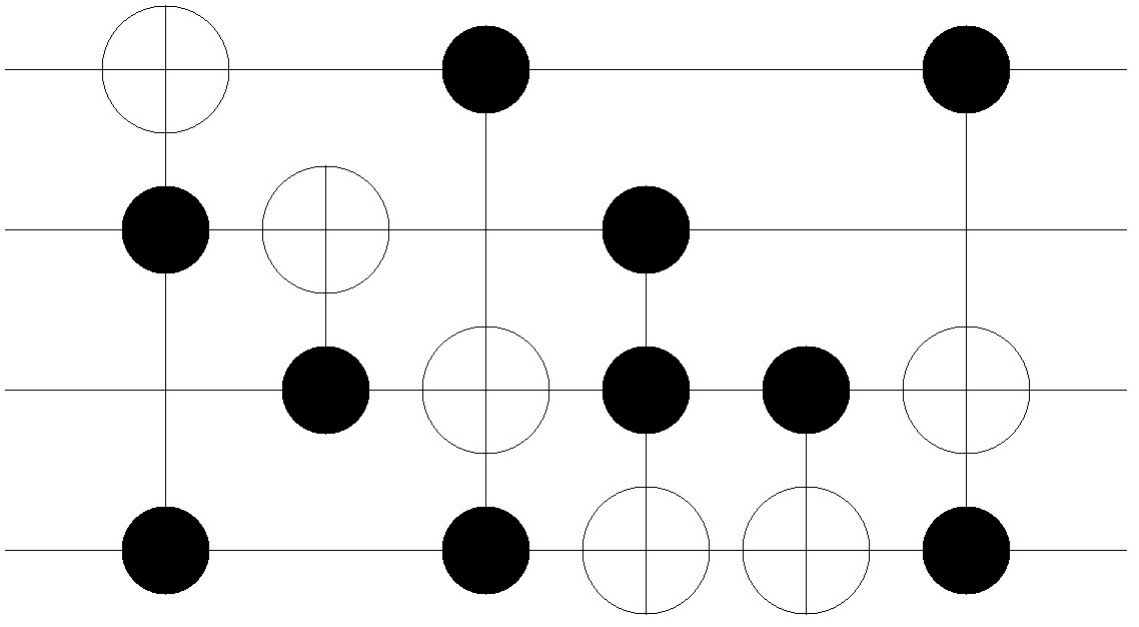
Resulting circuits of No. 18 *mini_alu* with varying *N*_*D*_: *N*_*D*_ = 6.

**Fig 13:**
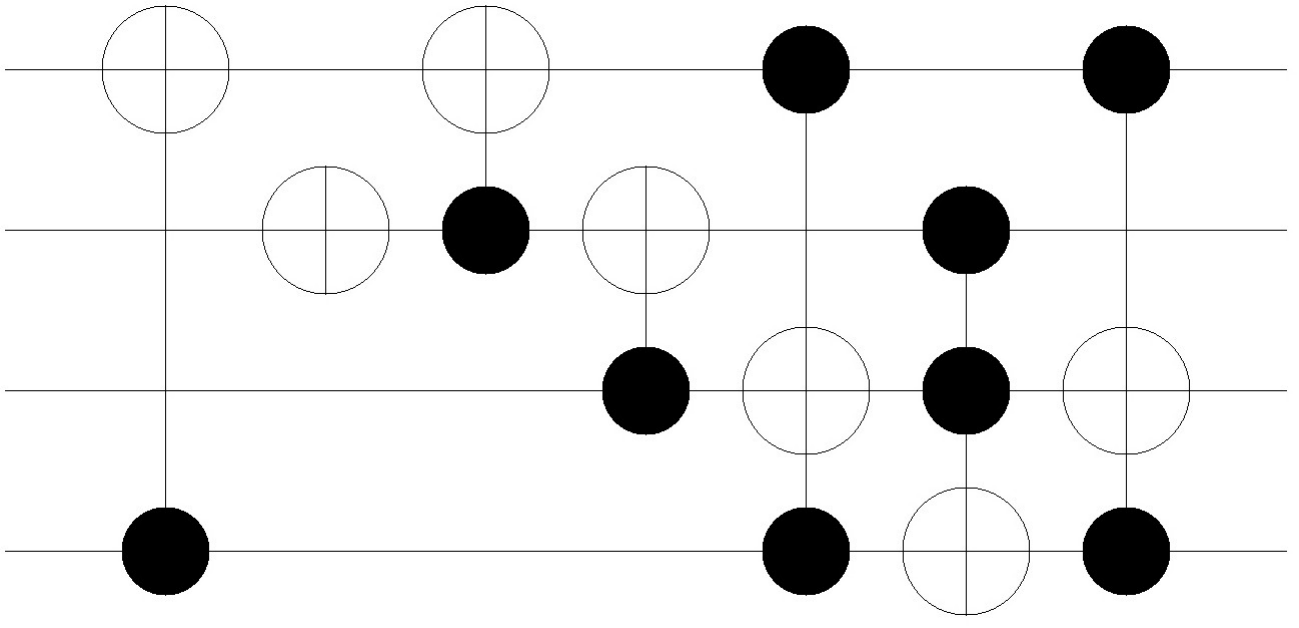
Resulting circuits of No. 18 *mini_alu* with varying *N*_*D*_: *N*_*D*_ = 7.

**Fig 14:**
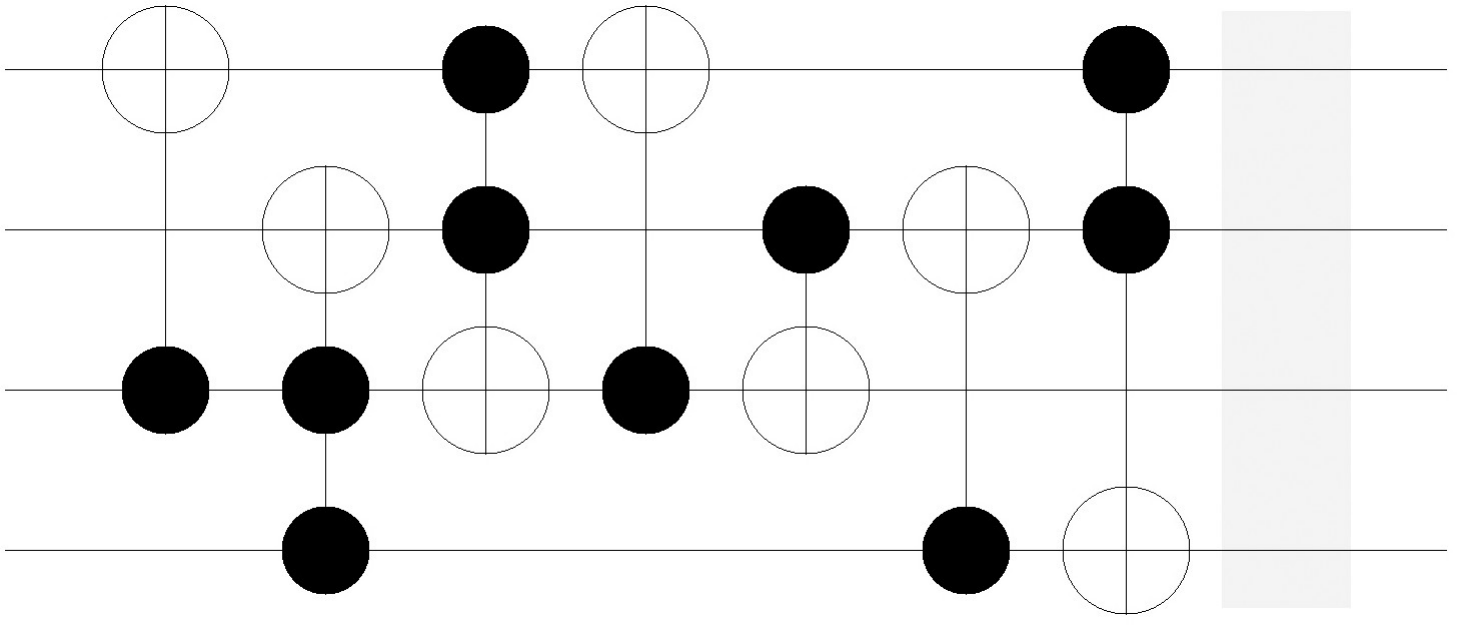
Resulting circuits of No. 18 *mini_alu* with varying *N*_*D*_: *N*_*D*_ = 8.

**Fig 15:**
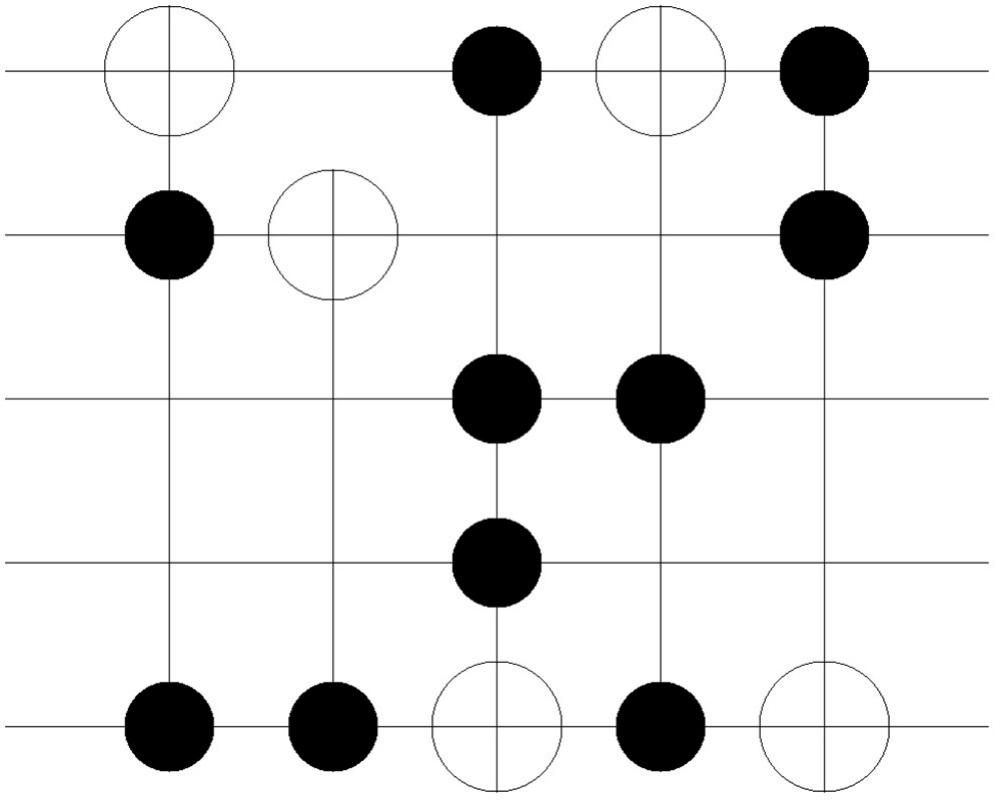
Resulting circuits of No. 18 *4gt4_v1* with varying *N*_*D*_: *N*_*D*_ = 5.

**Fig 16:**
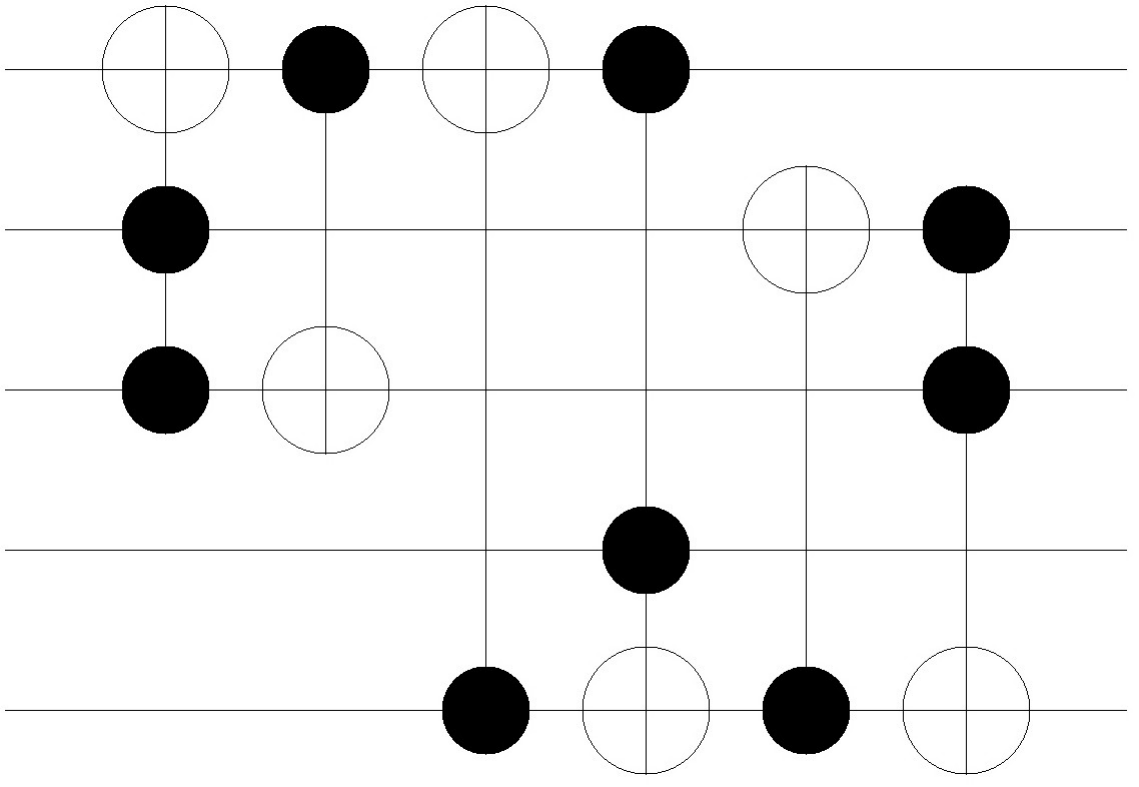
Resulting circuits of No. 18 *4gt4_v1* with varying *N*_*D*_: *N*_*D*_ = 6.

**Fig 17:**
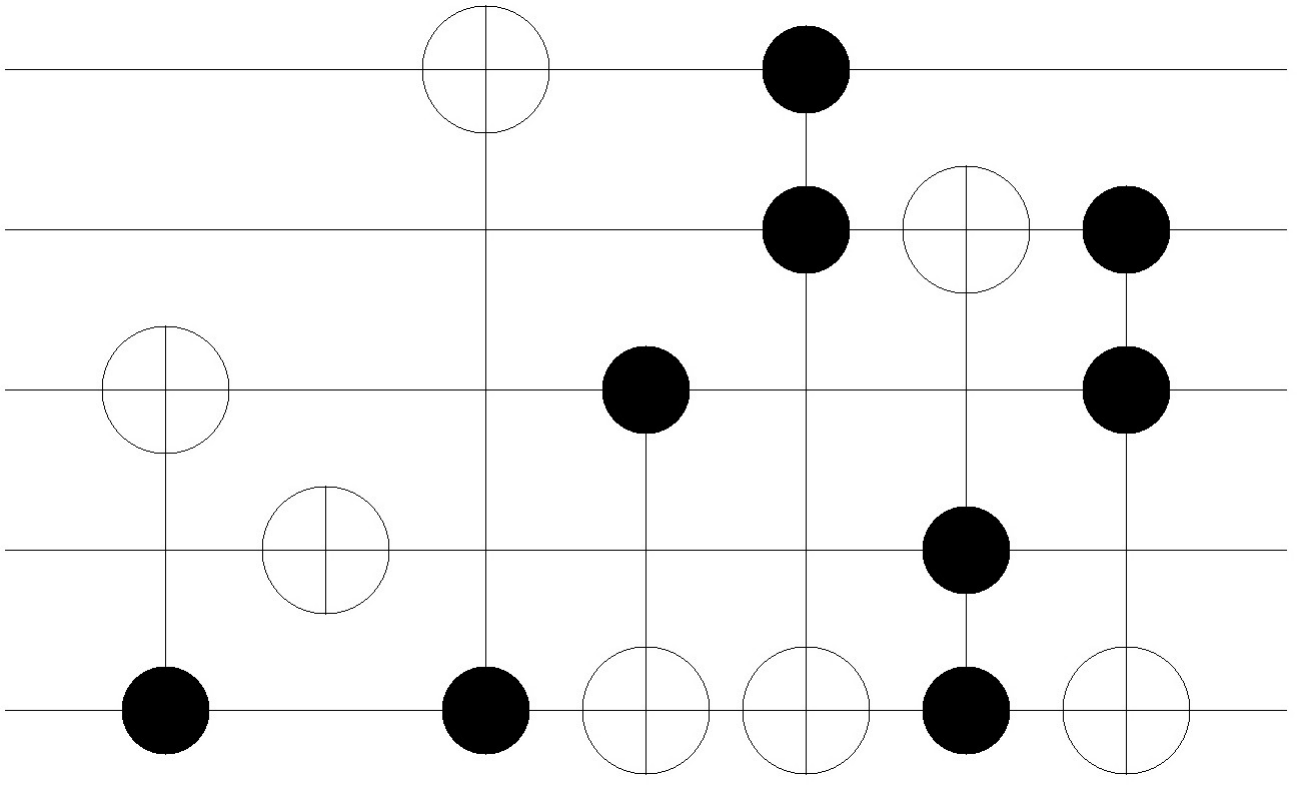
Resulting circuits of No. 18 *4gt4_v1* with varying *N*_*D*_: *N*_*D*_ = 7.

**Fig 18:**
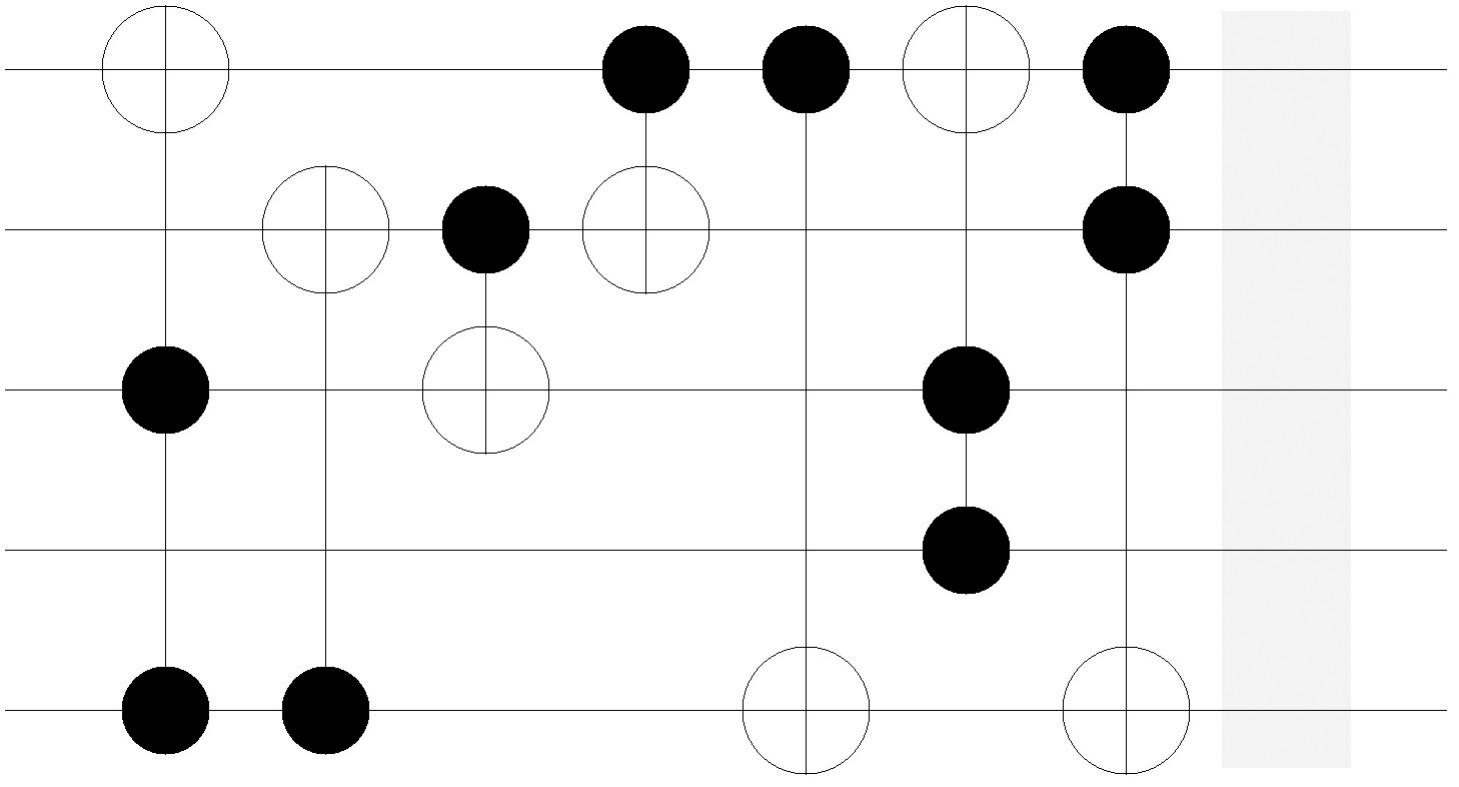
Resulting circuits of No. 18 *4gt4_v1* with varying *N*_*D*_: *N*_*D*_ = 8.

The improvement in the quantum costs is also shown in the resulting circuit. Figs 11–18 show the solution obtained in a circuit composed of MCT gates. The four circuits in Figs 11–14 are the result of No. 18 *mini_alu*, whereas the circuits in Figs 15–18 denote the circuit implementing the function No. 26 *4gt4_v1*. As mentioned in Section 2, the more control bits an MCT gate includes, the higher the quantum cost that is required to implement the gate physically. In Fig 11, the circuit is composed of five *C*^2^ *NOT* gates. However, in the following Figs 12–14, the number of *C*^2^ *NOT* gates decreases, and *CNOT* gates are used instead. The same trend is also shown in Figs 15–18. Although the *C*^3^ *NOT* gate is shown in Fig 15, the circuits in Figs 16–18 are composed of *CNOT* and *C*^2^ *NOT* gates. Thus, quantum costs decrease as the value of *N*_*D*_ increases in both cases.

## Discussions

We propose a network-based optimization model that synthesizes an optimal cost circuit for frequently used Boolean reversible functions in quantum computing. Notably, the optimization model for the QRCS problem appears uniquely as an extended form of the MCNF model that charges the network cost according to the subset of arcs selected. We also present experimental results based on benchmark data. In particular, comparing the experimental results with prior studies, we obtain improved solutions in terms of quantum cost for almost all of our data. The improvements in quantum costs have occurred from a minimum of 18.8% to a maximum of 68.6%.

The two main contributions of our research are as follows. First, our work suggests a novel research perspective to both a mathematical optimization and the quantum computing field. From a mathematical optimization perspective, this study proposes an exciting application of optimization to the practical problems arising from this new technical field. In addition, the optimization-based approach enhances the robustness and practicality of a quantum reversible circuit synthesis from the perspective of quantum computing. We introduce a realistic objective function that minimizes the number of fundamental quantum gates required when implementing Boolean reversible functions. Our approach can also be utilized to evaluate the heuristic algorithms fora circuit synthesis because the optimality of the solution is guaranteed. The optimal Boolean reversible circuits developed through the proposed model can also be used as building blocks for the later synthesis of large-size reversible circuits.

Second, we propose a new expansion of the MCNF model that uniquely appears in the QRCS problem. In contrast to the conventional MCNF model that charges the network cost per arc, the extended version sets the network cost according to the selected subset of arcs in each level. The proposed model also shows significantly better experimental results regarding both the solution quality, computational time, and optimality gap. Owing to the increased tractability of the newly proposed model, we conducted experiments within a broader range of the maximum number of gates. The results empirically show that the overall quantum cost required to implement a Boolean reversible circuit decreases when a larger number of MCT gates are used.

However, the experimental results show that when the size of the problem increases, the optimality gap does not sufficiently narrow within maximum calculation time. Therefore, follow-up research is underway to develop an optimization methodology that can handle large-scale problems. We have initiated this research by selecting an optimization methodology suitable for the proposed model structure. Furthermore, to efficiently solve the model, studies on heuristic algorithms are being planned. Future research on an extended MCNF model is also required and is expected to have potential applicability to various other conventional problems such as distribution and time-stage network problems, among others.
